# Modest Interference with Actin Dynamics in Primary T Cell Activation by Antigen Presenting Cells Preferentially Affects Lamellal Signaling

**DOI:** 10.1371/journal.pone.0133231

**Published:** 2015-08-03

**Authors:** Kole T. Roybal, Emily M. Mace, Danielle J. Clark, Alan D. Leard, Andrew Herman, Paul Verkade, Jordan S. Orange, Christoph Wülfing

**Affiliations:** 1 Department of Immunology, UT Southwestern Medical Center, Dallas, Texas, United States of America; 2 Children's Hospital of Philadelphia Abramson Research Center, University of Pennsylvania, Philadelphia, Pennsylvania, United States of America; 3 School of Cellular and Molecular Medicine, University of Bristol, Bristol, United Kingdom; 4 School of Biochemistry, University of Bristol, Bristol, United Kingdom; 5 Department of Cell Biology, UT Southwestern Medical Center, Dallas, Texas, United States of America; University of Iowa, UNITED STATES

## Abstract

Dynamic subcellular distributions of signaling system components are critical regulators of cellular signal transduction through their control of molecular interactions. Understanding how signaling activity depends on such distributions and the cellular structures driving them is required for comprehensive insight into signal transduction. In the activation of primary murine T cells by antigen presenting cells (APC) signaling intermediates associate with various subcellular structures, prominently a transient, wide, and actin-associated lamellum extending from an interdigitated T cell:APC interface several micrometers into the T cell. While actin dynamics are well established as general regulators of cellular organization, their role in controlling signaling organization in primary T cell:APC couples and the specific cellular structures driving it is unresolved. Using modest interference with actin dynamics with a low concentration of Jasplakinolide as corroborated by costimulation blockade we show that T cell actin preferentially controls lamellal signaling localization and activity leading downstream to calcium signaling. Lamellal localization repeatedly related to efficient T cell function. This suggests that the transient lamellal actin matrix regulates T cell signaling associations that facilitate T cell activation.

## Introduction

T cells activate in cellular interactions with antigen-presenting cells (APC). During activation, the T cell signaling system displays a distinct spatiotemporal organization [1–5]. Yet, it is largely unresolved how the dynamic spatiotemporal organization of T cell signaling contributes to signaling activity. The reciprocal connection between signaling organization and function has been extensively studied where the initiation of signaling and the cellular process regulated by it share a subcellular location, e.g. in phagocytosis [6] and secretory granule release [7]. However, it has remained largely elusive how signaling activity can be controlled by regulating the cell-wide spatiotemporal organization of an entire signaling system. Here we characterize the role of actin dynamics in the organization of T cell signaling as it relates to function.

Some elements of the complex and dynamic spatiotemporal organization of T cell activation on APCs [3] are long established, particularly the accumulation of molecules at the interface center (TCR, PKCθ) and in the periphery (LFA-1, actin) [1, 2, 8]. We have recently extended the investigation of signaling distributions in the activation of primary T cells by APCs to more than 60 signaling intermediates (accompanying manuscript). A dominant localization amongst various stereotypical signaling distributions is accumulation in a wide, transient, and actin-associated lamellum extending from an undulating T cell:APC interface several μm deep into the T cell. As subcellular signaling distributions control the efficiency of molecular signaling interactions, their regulation is of substantial interest.

Actin dynamics are crucial general regulators of cellular organization. In T cells, interference with actin regulation has established that actin dynamics are critical for T cell activation, e.g. in the regulation of cell conjugate formation, receptor clustering, lytic granule release, calcium signaling, and activation of transcription factors [9–12]. Because of superior experimental access, the spatiotemporal organization of T cell signaling and its regulation by actin dynamics have been predominantly studied using planar APC substitutes [13–19]. By investigating cellular organization in primary T cells activated by APCs at the systems scale, it has recently become apparent that the spatiotemporal organization of T cell signaling and the cell biological structures driving it differ between T cells activated with APCs and planar substitutes thereof (accompanying manuscript). Therefore the question of how actin regulates signaling organization and activity in primary T cell:APC couples is largely unresolved.

Here we use modest pharmacological interference with actin dynamics and blockade of costimulation, a well-know regulator of actin in T cells, to investigate how various signaling distributions depend on actin. We show that actin regulates signaling organization and activity associated with a wide, transient, and actin-associated lamellum extending from an undulating T cell:APC interface several μm deep into the T cell. Disruption of lamellal signaling leads to altered activity of key T cell signaling components and calcium signaling.

## Results

### A low concentration of Jasplakinolide modestly impairs interface actin accumulation with a lamellal preference while leaving cell coupling intact

In our investigation of the role of actin dynamics in signaling organization and activity, we imaged T cell signaling via live cell fluorescence microscopy at a large scale. *In vitro* primed primary 5C.C7 TCR transgenic CD4^+^ T cells were retrovirally transduced to express fluorescently tagged signaling intermediates and sensors. Time-lapsed fluorescence microscopy was performed with transduced T cells activated by CH27 B cell lymphoma APCs pulsed with 10μM moth cytochrome C (MCC) antigenic peptide. This experimental setup provides an *in vitro* model for the reactivation of primed T cells, e.g. in the delivery of T cell help.

**Fig 1:**
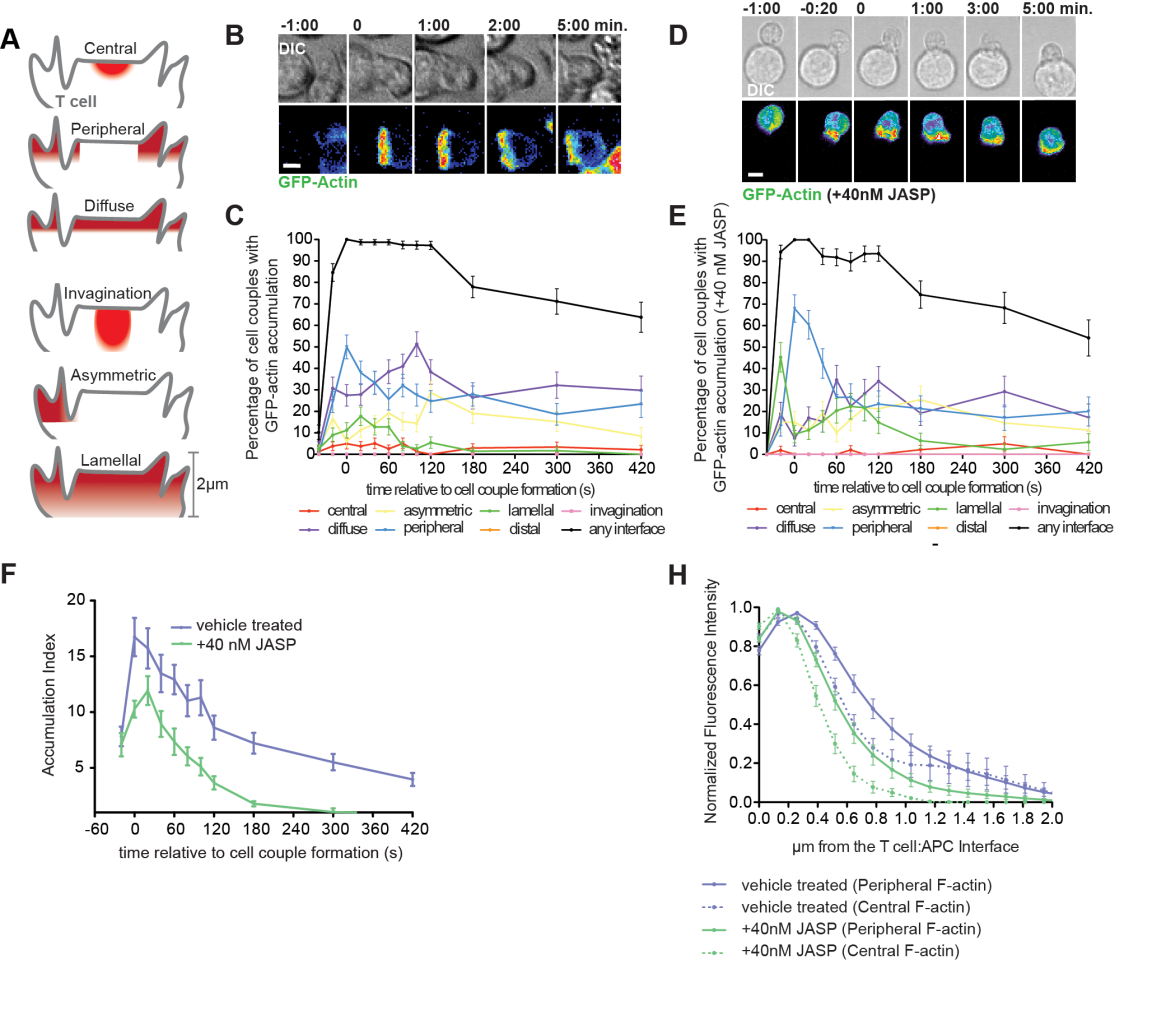
Low dose JASP treatment only modestly interferes with interface actin accumulation. **(A)** Principal actin and signaling distributions are schematically represented: The APC above the T cell is not shown. Central reflects a large central signaling complex, peripheral the part of the actin network stabilizing the interface edge. Diffuse reflects cortical accumulation, invagination enrichment in a transient large T cell invagination suggested to contribute to early signal resetting [20]. Asymmetric reflects individual small membrane extensions and the lamellal pattern represents accumulation in a large transient actin sheet (‘lamellum’) extending from the undulating T cell:APC interface μm deep into the T cell, as characterized in detail in an accompanying manuscript. **(B, D)** Representative interactions of 5C.C7 T cells expressing GFP-actin with peptide-loaded CH27 APCs (10μM MCC) over the indicated time relative to formation of a tight cell conjugate are given in control T cells (B) and T cells treated with 40nM Jasplakinolide (D). DIC images are shown on top and top-down maximum projections of 3D fluorescence data are shown in the bottom panels in a rainbow-like, false-color intensity scale (increasing from blue to red)(scale bar = 5μm). (**C, E**) 5C.C7 T cells expressing GFP-actin were stimulated with peptide loaded CH27 APCs (10μM MCC). The percentage of T cells showing accumulation in defined patterns [3] among all cell couples analyzed across multiple experiments as schematically represented in (A) is shown under the (C) control (number of cell couples analyzed across multiple independent experiments, n = 80) or (E) 40nM Jasplakinolide (n = 53) condition. (**F**) 5C.C7 T cells expressing GFP-actin were stimulated with peptide loaded CH27 APCs (10μM MCC) in the presence of 40nM Jasplakinolide (n = 25) or DMSO (n = 25). The accumulation index, a measure for the amount of GFP-actin recruited to the cellular interface, is plotted relative to the time of tight cell conjugate formation. (**G**) GFP-actin amounts relative to maximum as a function of the distance from the interface were measured from the same live cell 5C.C7:CH27 conjugates as in C, E. The measurements were made separately for the center (delineated by the 25% and 75% marks across the interface diameter) and periphery (remainder) of the interface and are shown accordingly. Control data are the same as in Fig 2C of the accompanying manuscript.

It is well established that gross interference with actin dynamics impairs various aspects of T cell activation [9–12]. However, in the activation of primary T cells by APCs, the mechanism and extent of actin regulation of T cell signaling remains largely unresolved. To address this question, an actin interference regime has to be found that leaves principal elements of T cell activation, cell coupling and the formation of a wide cellular interface intact. In addition, changes to actin distributions at the T cell:APC interface should focus on defined elements thereof. In primary T cell:APC couples, actin forms a ring around the periphery of the T cell:APC interface, actin underlies individual asymmetric membrane extensions, cortical actin aligns with the plasma membrane, and a large transient actin sheet (‘lamellum’) extends from the undulating T cell:APC interface deep into the T cell (Fig 1A)(accompanying manuscript). In devising an actin interference regimen, a large number of molecular regulators of T cell actin are well characterized [9]. However, many of them directly influence signaling, and adjusting their expression levels for the necessary modest extent of actin interference is exceedingly difficult. Therefore, we used drugs that directly target actin in a carefully titrated fashion instead.

We achieved the desired modest interference with T cell actin using a low dose, 40nM, of Jasplakinolide (henceforth referred to as low dose JASP treatment), a drug that while stabilizing F-actin often leads to diminished F-actin structures in live cells [21](Fig 1B–1G). As the foundation of this study, we extensively characterized the changes to actin and actin-dependent cellular processes upon low dose JASP treatment first.

Importantly, principal elements of T cell actin dynamics, cell coupling, a wide interface, and the geometry of actin distributions, remained largely intact upon low dose JASP treatment: 44±5% of T cells contacting an APC still proceeded to form a tight cell couple, only slightly reduced compared to cell coupling frequencies in the absence of drug of 60±4% [22, 23](S1A Fig). Increasing the Jasplakinolide concentration to 100nM reduced cell coupling significantly (8±6%, p = 0.02 compared to 40nM); 500nM Jasplakinolide caused rapid apoptosis of the primary 5C.C7 T cells. Upon low dose JASP treatment the T cell:APC interface was as wide as under control conditions (5.6±0.3μm control versus 5.9±0.3μm low dose JASP within 2min of cell coupling as determined by EM). Spatiotemporal patterning of actin was only marginally altered (Fig 1B–1E, S1 Video). The only significant change occurred during the initial rapid spreading of a fraction of actin from an initial accumulation across the entire interface to the edge of the interface. While such spreading was sufficiently fast under control conditions that a separate time point with preferential actin accumulation across the entire interface in either the lamellal or diffuse pattern could not be detected, upon low dose JASP treatment diffuse and lamellal patterning was dominant at the -20 time point (64±7% diffuse plus lamellal patterning upon low dose JASP treatment versus 40±6% under control, p = 0.01). This change is consistent with modestly slowed actin dynamics, as supported by fluorescence recovery after photobleaching (FRAP) experiments (S1B Fig).

**Fig 2:**
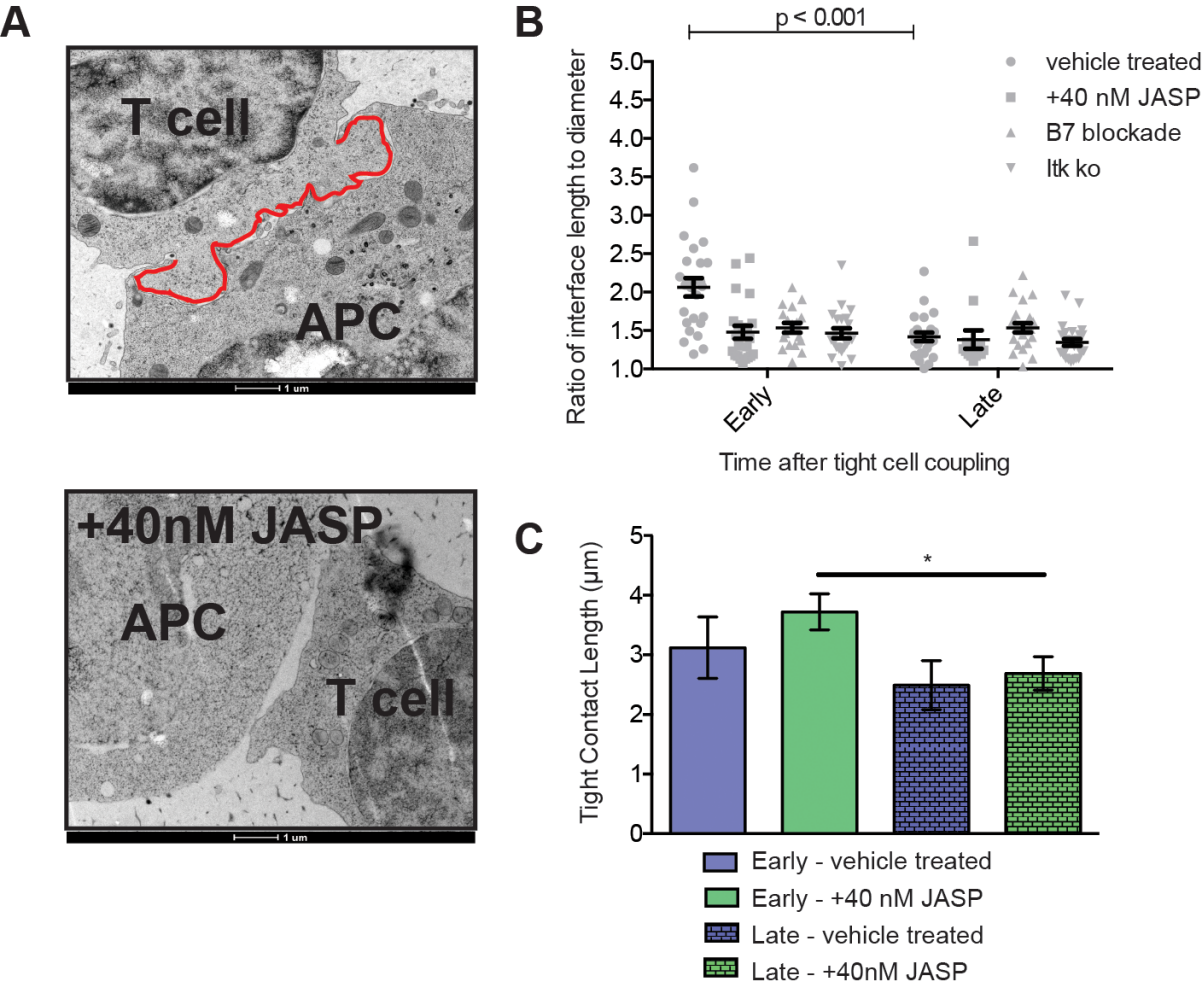
Low dose JASP treatment inhibits the formation of early T cell interface membrane undulations. (**A**) Representative electron micrographs of 5C.C7:CH27 conjugates at an early time point as control (top) and treated with 40nM Jasplakinolide (bottom) are given. In the control image the interface part of the T cell membrane is highlighted in red to illustrate the membrane undulations. The control image is from the same cell couples as Fig 3B in the accompanying manuscript. (**B**) 5C.C7 T cells were activated with peptide-loaded CH27 APCs (10μM MCC) and processed for EM at an early and late time point as detailed in the methods section. The interface length to diameter ratio is given for four conditions: control 5C.C7 T cells (number of cell couples analyzed across multiple independent experiments, n = 51), 5C.C7 T cells treated with 40nM Jasplakinolide (n = 35), Itk-deficient 5C.C7 T cells (n = 55), 5C.C7 T cells activated by CH27 APCs upon blockade of B7-1/2 (n = 41). **(C)** Lengths of tight interface contact between at the T cell:APC (i.e. regions of opposing T cell:APC membrane <20nm apart rather than the entire interface outline denoted as ‘interface length’) are given from some of the same electron micrographs analyzed for B. Error bars are s.e.m.. Significance was determined by Student’s t-test and is indicated by an asterisk (*p<0.05).

Other elements of actin dynamics, however, were more severely impaired upon low dose JASP treatment. Interface actin amounts were reduced (Fig 1F). The extent of actin reaching into the T cell away from the interface across the entire width of the interface, a key feature of lamellal actin, was diminished (Fig 1G). As recently characterized using superresolution and electron microscopy (accompanying manuscript), F-actin structures perpendicular to the T cell:APC interface are similar in character to the dramatic interface undulations revealed by EM (Fig 2A). Upon low dose JASP treatment, membrane undulations at the T cell:APC interface were dramatically impaired: low dose JASP treatment reduced the interface length to diameter ratio from 2.1±0.2 to 1.5±0.2 (p<0.05) at an early time point (≤ 2min)(Fig 2B). Interestingly, as the length of tight T cell:APC contact, i.e. the part of the T cell:APC interface with a membrane distance that can be spanned by the TCR and similarly sized receptors, was not substantially altered (Fig 2C), receptor ligand binding is unlikely to differ.

In summary, low dose JASP treatment left principal features of actin dynamics largely intact, but thinned the actin matrix, reduced the extent actin reaches from the interface into the T cell, and led to loss of actin-associated early membrane undulations. Importantly, all three phenotypes are strongly related to lamellal actin rather than actin accumulation in peripheral, cortical, or single membrane extension distributions. The data suggest that low dose JASP treatment preferentially diminishes lamellal actin.

### Low dose JASP treatment preferentially alters lamellal signaling localization

With the low dose JASP treatment, we could now investigate roles of actin dynamics in signaling organization and activity. A powerful way to do so is to assess the consequences of actin interference, not on one, but on a group of signaling intermediates that collectively represent critical elements of signaling organization. Key elements of signaling organization in primary T cells activated by APCs have been recently reviewed [24], are summarized in Fig 1A, and more than 60 signaling intermediates have been recently mapped onto cellular structures (accompanying manuscript). These structures include a central signaling complex [1, 3, 5, 25], a transient invagination [20], cortical accumulation, and enrichment at the interface periphery [1] and in the wide transient actin lamellum. First, we investigated a number of lamellal signaling intermediates. SLP-76, PIP_2_, Themis, and Vav1 all displayed strong lamellal localization. However, SLP-76 was initially centrally localized and Vav1 showed strong peripheral localization in addition to lamellal. As a comparison, LAT and PKCθ represent central signaling [24].

**Fig 3:**
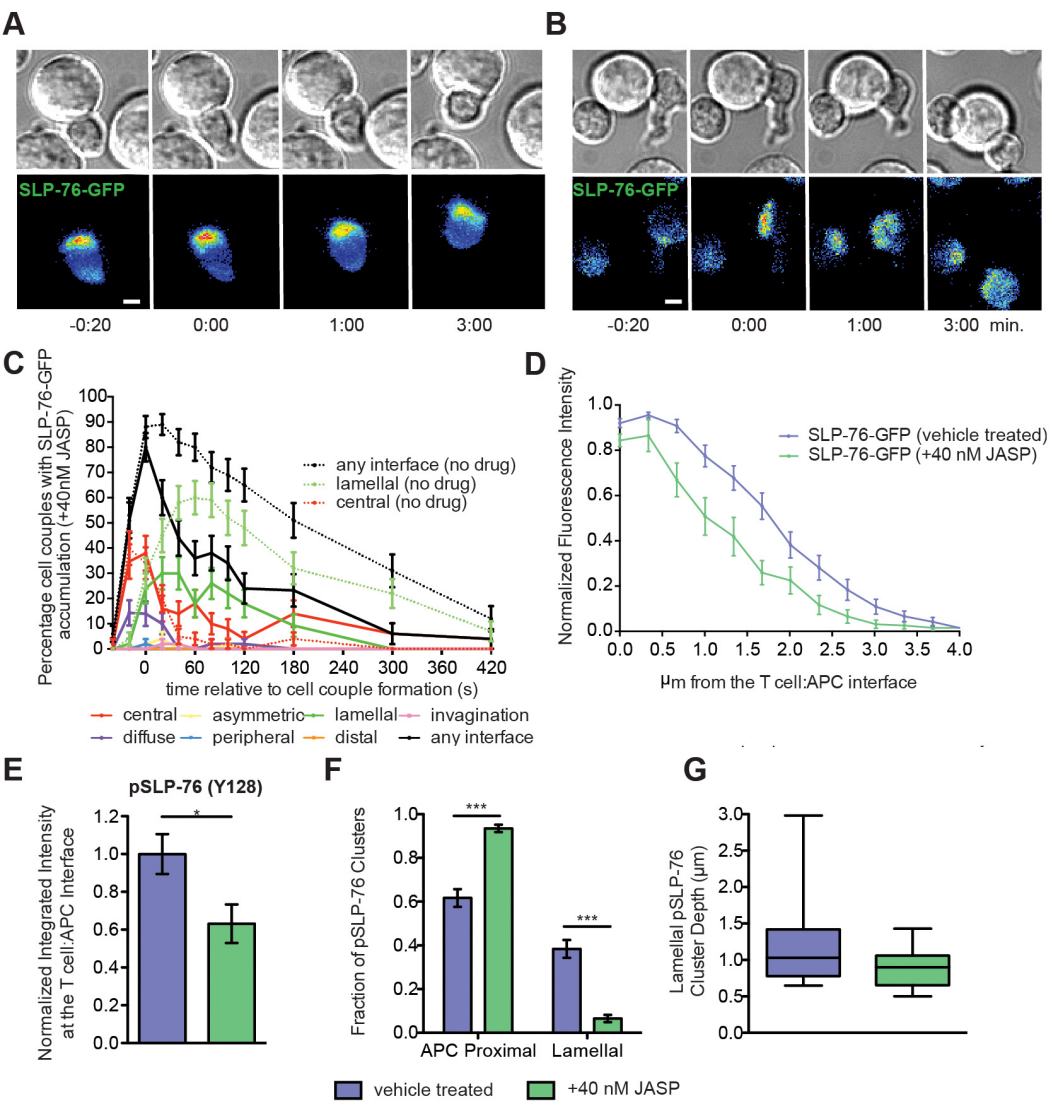
Low dose JASP treatment interferes with lamellal SLP-76 localization. **(A, B)** Representative interactions of 5C.C7 T cells expressing SLP-76-GFP with peptide-loaded CH27 APCs (10μM MCC) under control conditions (A) or in the presence of 40nM Jasplakinolide **(** S2 Video)(B) over the indicated time relative to formation of a tight cell conjugate is given as in Fig 1B. The control data are a reproduction of Fig 1B in the accompanying manuscript. (**C**) The corresponding pattern classification is given upon treatment with 40nM Jasplakinolide similar to Fig 1D (number of cell couples analyzed across multiple independent experiments, n = 50). Control ‘any interface’, ‘central’ and ‘lamellal’ accumulation curves from Fig 1E in the accompanying manuscript are plotted as a reference in broken lines. **(D)** 5C.C7 T cells expressing SLP-76-GFP were activated with peptide-loaded CH27 APCs (10μM MCC). The fluorescence intensity relative to maximum as a function of the distance from the interface, measured from live cell conjugates upon control (n = 19) and low dose JASP treatment (n = 15) at 1min across the entire interface, is given. Control data are from Fig 1G of the accompanying manuscript. **(E**) 5C.C7 T cell:CH27 APC conjugates under control conditions or treated with 40nM Jasplakinolide were fixed at the 2min time point and stained for F-actin (Phalloidin), pSLP-76 (Y128), and APCs with Cell Trace Violet (representative images are in Fig 4A and 4B). pSLP-76 (Y128) cluster intensity is given for DMSO control (n = 17) and low dose JASP (n = 17) treated cell conjugates. (**F**) Analyzing the same cell couples as in E, the frequencies of APC proximal (Fig 4B) and lamellal (Fig 4A) clusters are given. (**G**) Analyzing the same cell couples as in E, the distance of lamellal pSLP-76 clusters from the interface for DMSO control (32 clusters) and residual lamellal clusters in low dose JASP treated cell conjugates (12 clusters) is plotted (whiskers = min and max). Significance was determined by Student’s t-test and is indicated by asterisks (*p<0.05, ***p<0.001).

**Fig 4:**
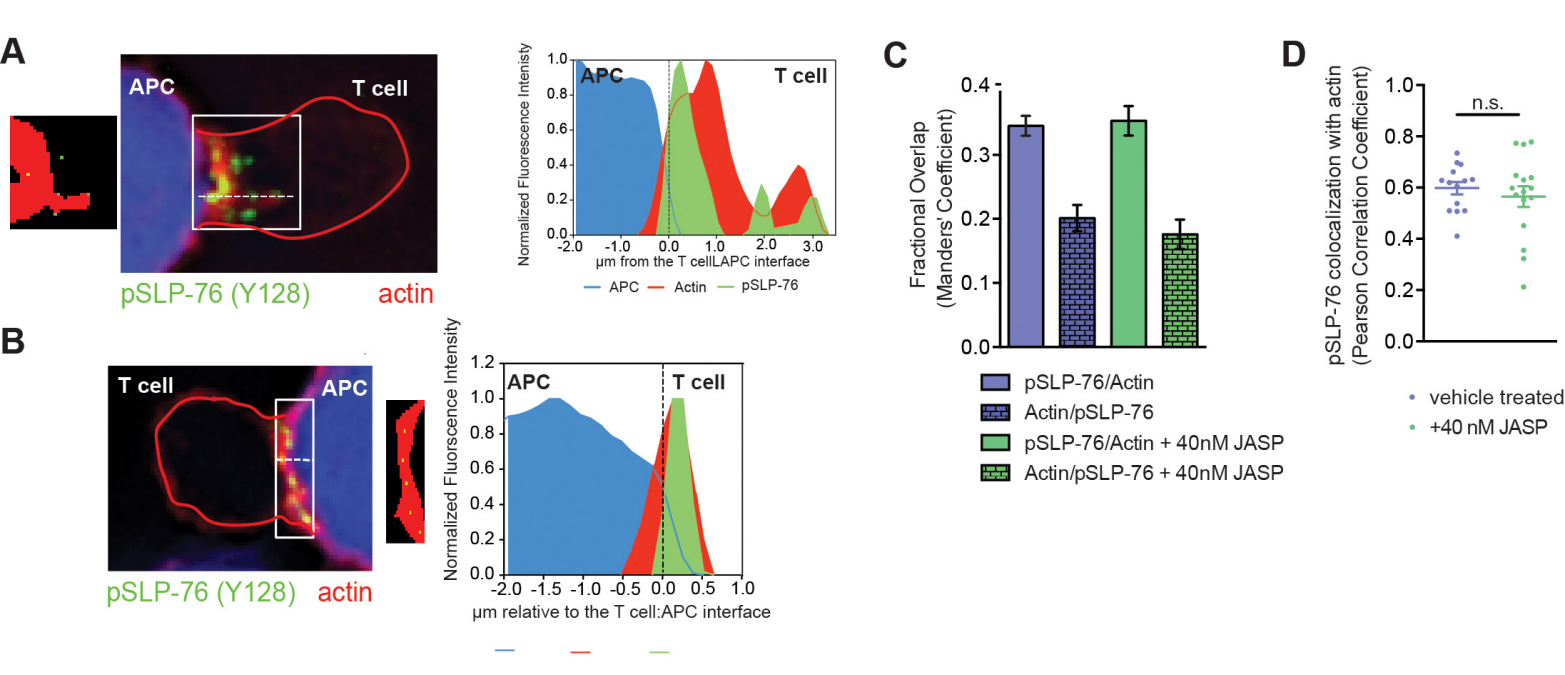
Residual pSLP-76 remains co-localized with actin upon low dose JASP treatment. (**A, B**) 5C.C7 T cell:CH27 APC conjugates under control conditions (A) or treated with 40nM Jasplakinolide (B) were stained for F-actin (Phalloidin), pSLP-76 (Y128), and APCs with Cell Trace Violet. The T cell outline is denoted in red. Representative images are shown on the left while intensity line scans perpendicular to the interface (dotted white line) are shown on the right. Binary masks of above background F-actin (red) are shown with the centers of mass of pSLP-76 clusters (green) next to the representative images. The clusters in the low dose JASP-treated T cell are representative of ‘APC proximal’ clusters, the clusters in the control T cell of ‘lamellal’ clusters. A is the same as Fig 6B in the accompanying manuscript. (**C**) Analyzing the same cell couples as in Fig 3E, Mander’s coefficient reporting the percentage of pSLP-76 that overlaps with F-actin and vise versa is given. Control data are the same as in Fig 3F of the accompanying manuscript. (**D**) Analyzing the same cell couples as in Fig 3E, Pearson’s correlation coefficients for colocalization of pSLP-76 with F-actin is given. Control data are the same as in Fig 3H of the accompanying manuscript. Error bars are s.e.m..

**Fig 5:**
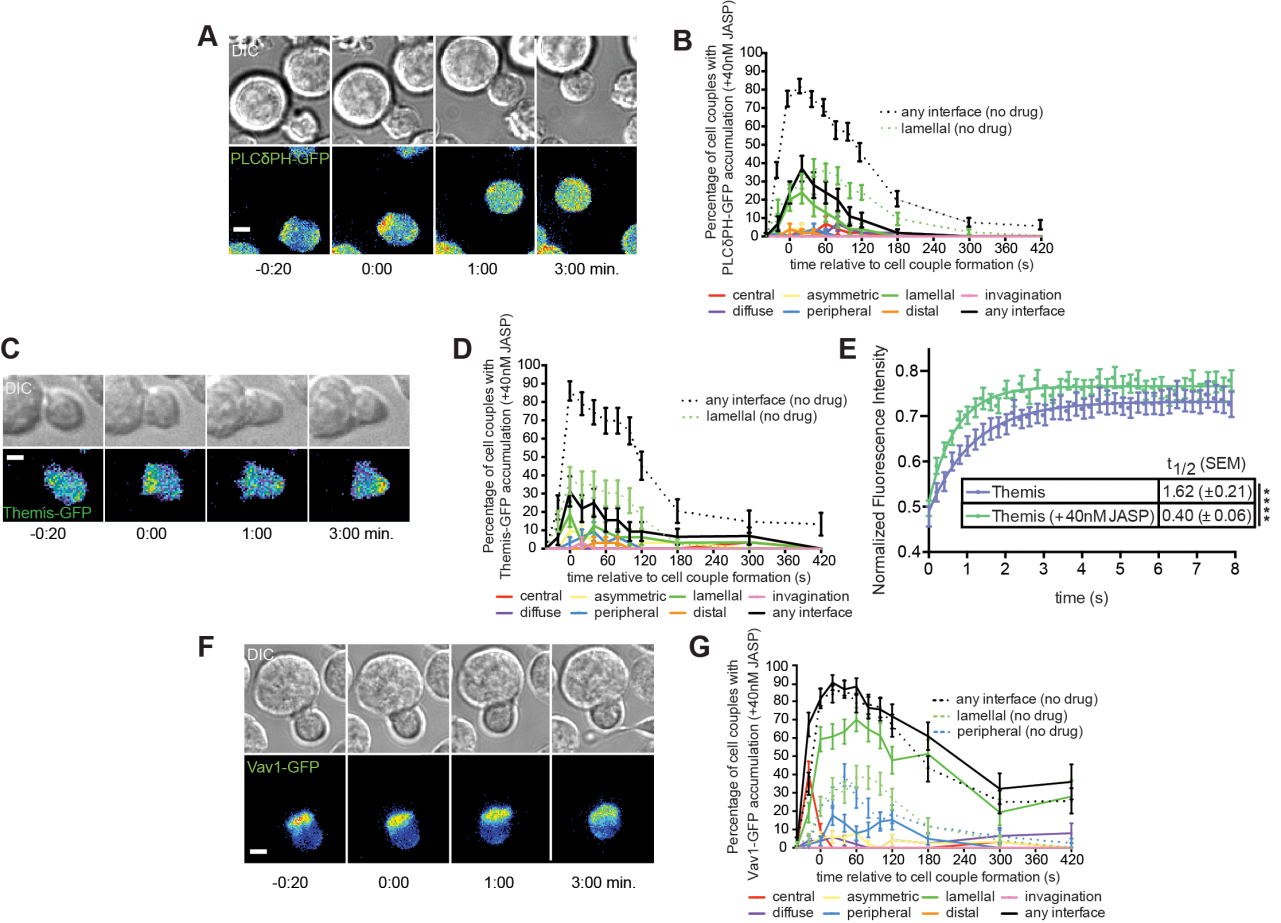
Low dose JASP treatment preferentially interferes with lamellal signaling localization. (**A**) A representative interaction of a 5C.C7 T cell expressing PLCδPH-GFP (PIP_2_ sensor) with 40nM Jasplakinolide (S3 Video **)** is given similar to Fig 1B. (**B**) The corresponding pattern classification is given similar to Fig 3C Control data are from Fig 1F in the accompanying manuscript. (number of cell couples analyzed across multiple independent experiments, n = 46). (**C**) A representative interaction of a 5C.C7 T cell expressing Themis-GFP with 40nM Jasplakinolide (S4 Video) is given similar to Fig 1B. (**D**) The corresponding pattern classification is given similar to B (n = 32)(control data from [26]). (**E)** 5C.C7 T cells expressing Themis-GFP were activated with CH27 APCs (10 μM MCC agonist peptide) and subjected to a FRAP experiment. Average fitted recovery curves and recovery half times are given for Themis with (n = 19) and without (n = 18) low dose JASP treatment. (**F**) A representative interaction of a 5C.C7 T cell expressing Vav1-GFP with 40nM Jasplakinolide (S5 Video) is given similar to Fig 1B. (**G**) The corresponding pattern classification is given similar to B upon addition of peripheral control data (from Fig 3C in [24])(n = 50). Error bars are s.e.m.. Significance was determined by Student’s t-test and is indicated by asterisks (****p<0.0001).

Low dose JASP treatment preferentially affected lamellal signaling localization. Low dose JASP treatment dramatically diminished the percentage of cell couples with lamellal localization of SLP-76 from 60±7% to 18±5% at 1min (p<0.0001) while leaving the preceding central accumulation intact. Overall recruitment of SLP-76 to the interface was more transient (Fig 3A–3C, S2 Video). The marginal amount of remaining SLP-76 accumulation was less deep (Fig 3D). pSLP-76 intensity at the T cell:APC interface as determined by fixed cell couple staining was reduced by 40% (p = 0.02) (Fig 3E). The percentage of pSLP-76 clusters localized deep into the lamellum was reduced from 38±4% to 7±2% (p<0.0001) (Fig 3F and 3G). Interestingly however, colocalization between active SLP-76 and F-actin in the residual areas of accumulation was not diminished (Fig 4). Low dose JASP treatment similarly diminished lamellal localization of PLCδPH and Themis (Fig 5A–5E, S3 and S4 Videos). For unknown reasons, the effect of low dose JASP treatment was more complex when a signaling intermediate associated with two F-actin-based patterns, lamellal and peripheral, rather than the lamellal pattern only: low dose JASP treatment enhanced the percentage of cell couples with lamellal localization of Vav1 from 39±6% to 70±6% (p = 0.0005) but at the expense of peripheral Vav1 recruitment (reduced from 27±6% to 8±4%, p = 0.02) (Fig 5F and 5G, S5 Video).

Lamellal signaling localization is accompanied by reduced mobility in FRAP assays (accompanying manuscript) suggesting that the lamellal actin matrix traps or binds associated signaling intermediates. Therefore we determined whether thinning lamellal actin with the low dose JASP treatment would release lamellal signaling intermediates. Indeed, low dose JASP treatment made the lamellal signaling intermediate Themis significantly more mobile in FRAP assays (t_1/2_ = 0.40±0.06s versus t_1/2_ = 1.62±0.21s, p<0.0001)(Fig 5E) with Themis reaching the mobility of GFP in non-treated cells (t_1/2_ = 0.32±0.04s).

**Fig 6:**
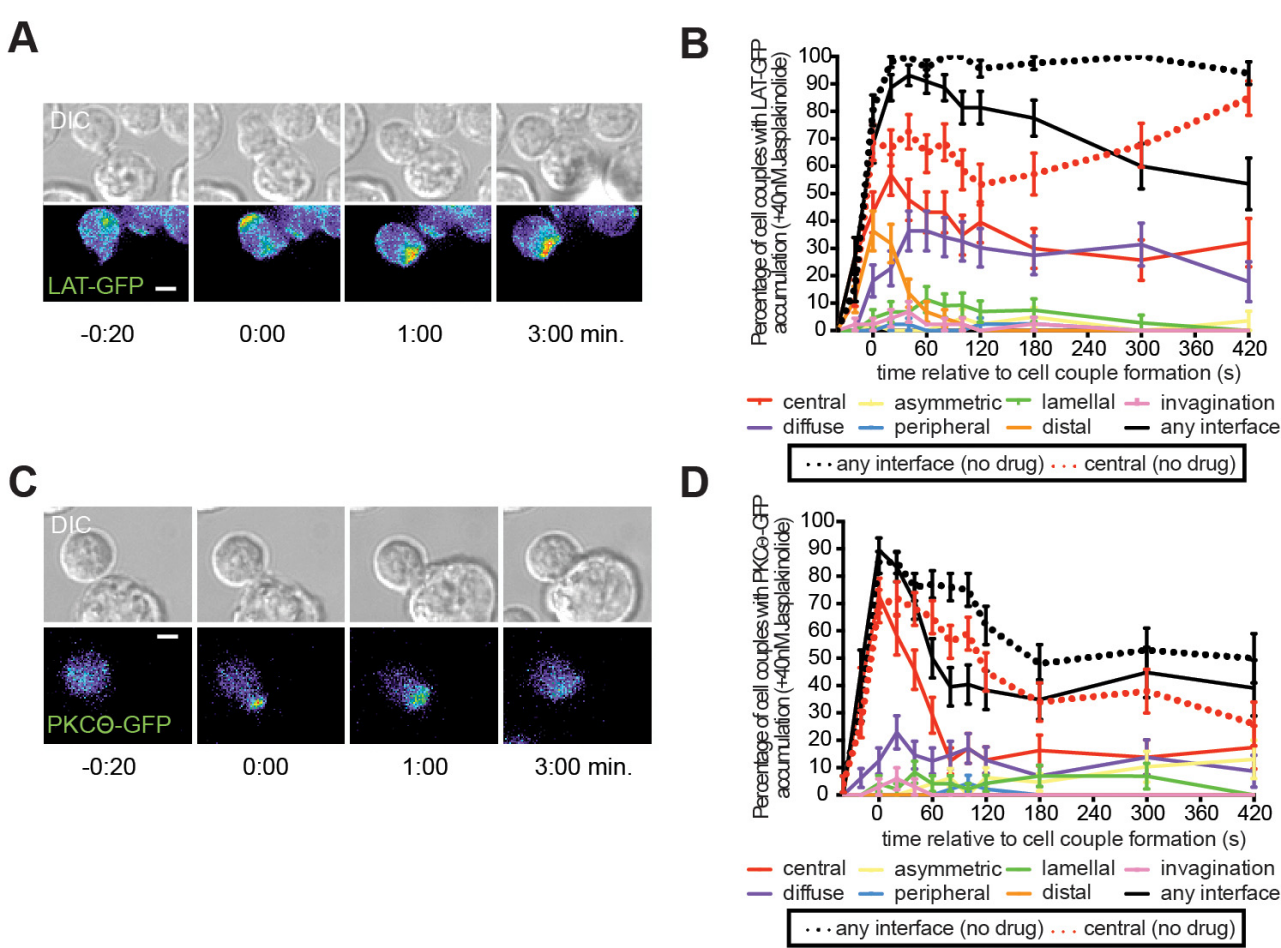
Low dose JASP treatment interferes less severely with central signaling localization. (**A, C**) Representative interactions of a 5C.C7 T cell expressing LAT-GFP (A) or PKCθ-GFP (C) with 40nM Jasplakinolide (S6 and S7 Videos) are given similar to Fig 1B. (**B, D**) The corresponding pattern classifications are given similar to Fig 3C (number of cell couples analyzed across multiple independent experiments, n = 44, n = 48). Control ‘any interface’ and ‘central’ accumulation curves [3] are plotted as a reference in broken lines. Error bars represent s.e.m..

In contrast to the strong actin dependence of lamellal signaling localization, central accumulation of LAT and PKCθ at the early time of lamellal patterning was more moderately impaired by low dose JASP treatment (Fig 6, S6 and S7 Videos). However, later sustained central localization was reduced by low dose JASP treatment, as not further pursued here.

In summary, low dose JASP treatment altered the localization of all signaling intermediates assayed, consistent with the expected role of actin as a general regulator of signaling organization. However, the extent of such alterations depended on the localization of the signaling intermediates. Lamellal signaling localization was most severely affected, consistent with the preferential interference of low dose JASP treatment with lamellal actin. The most straightforward explanation for this observation is that upon low dose JASP treatment, a less dense lamellal actin matrix binds or traps lamellal signaling intermediates less efficiently.

### Low dose JASP treatment impairs the activation of lamellal signaling intermediates

**Fig 7:**
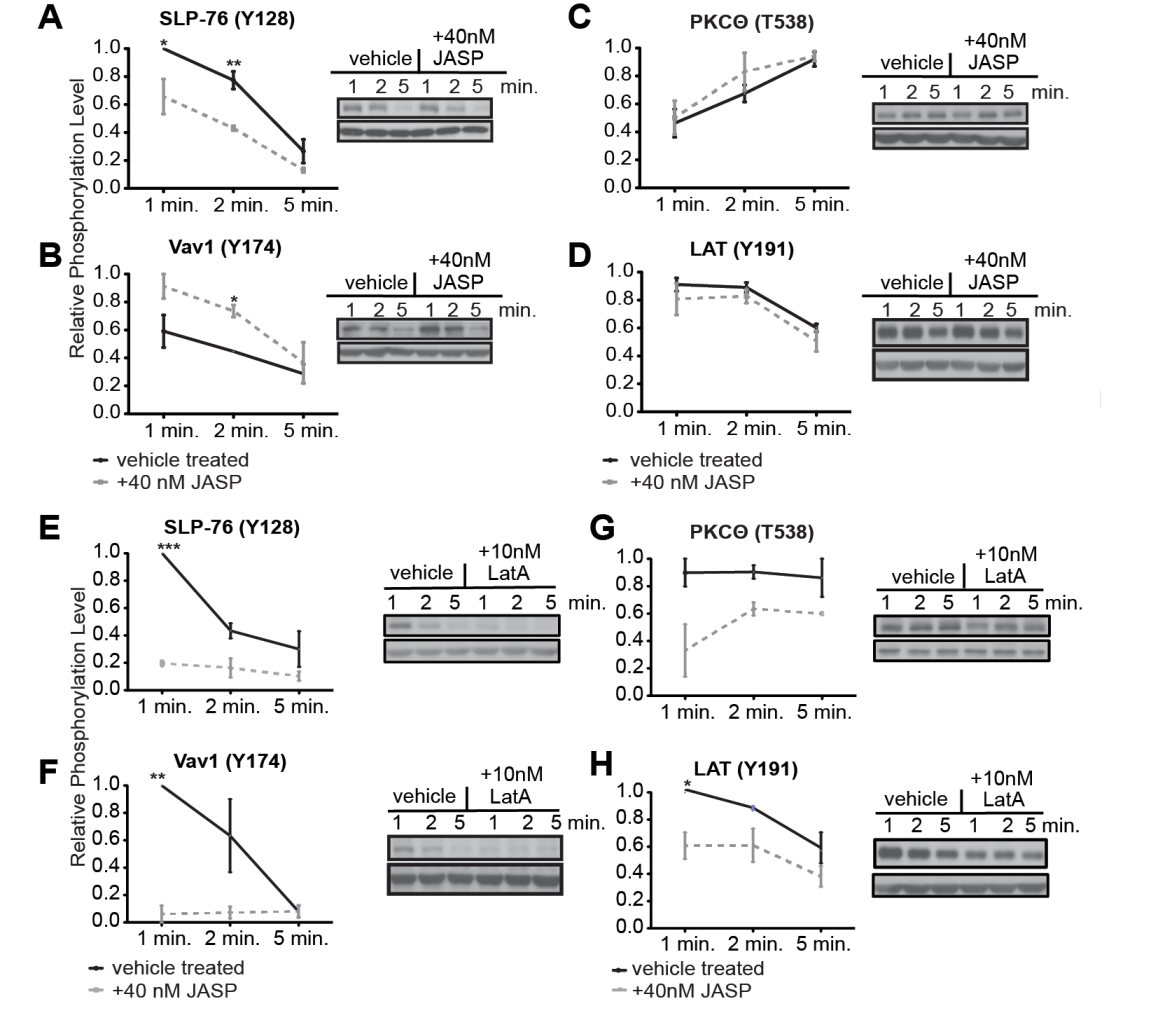
Low dose JASP treatment selectively affects lamellal signaling. (**A-D**) 5C.C7 T cells were activated with CH27 APCs (10μM MCC) with vehicle (solid black) or 40nM Jasplakinolide (broken grey). Phosphorylation levels for (A) SLP-76, (B) Vav1, (C) PKCθ, and (D) LAT were determined after 1, 2, and 5min. Graphs depict phosphorylation levels normalized to the max value jointly for control and Jasplakinolide treatment (at least 3 independent experiments). Corresponding representative immunoblots are adjacent to each graph. Complete blots are given in S2A–S2D Fig **(E-H)** 5C.C7 T cells were activated with CH27 APCs (10μM MCC) with vehicle (solid black) or 10nM LatA (broken grey). Phosphorylation levels for (E) SLP-76, (F) Vav1, (G) PKCθ, and (H) LAT were determined after 1, 2, and 5min and are given as in A-D. Complete blots are given in S2G–S2J Fig Error bar are s.e.m.. Significance was determined by Student’s t-test and is indicated by asterisks (*p<0.05,**p<0.001,***p<0.0001).

To establish whether altered localization of signaling intermediates affected their activity, we assessed their phosphorylation biochemically in 5C.C7 T cell:CH27 APC conjugates. Upon low dose JASP treatment, in parallel with the diminished lamellal SLP-76 localization (Fig 3C), whole cell pSLP-76 levels were reduced by 35±2% at 1min after tight cell coupling (p<0.05) and 34±9% at 2min (p<0.005) (Fig 7A), matching the 40% reduction in pSLP-76 intensity at the interface observed by microscopy (Fig 3E). Consistent with enhanced lamellal recruitment (Fig 5G), Vav1 showed a 30±4% increase in phosphorylation at 2min upon low dose JASP treatment (Fig 7B). Also, consistent with largely intact early central localization, LAT and PKCθ phosphorylation was unimpeded (Fig 7C and 7D). Taken together, these data strongly suggest that lamellal localization facilitates the activation of lamellal signaling intermediates. In contrast to low dose JASP treatment, more potent disruption of actin dynamics with 10nM Latrunculin A (LatA) reduced the activation of all proximal TCR signaling intermediates tested (Fig 7E–7H) while still allowing cell coupling and substantial interface actin accumulation (S2 Fig).

### Low dose JASP treatment selectively impairs calcium signaling

**Fig 8:**
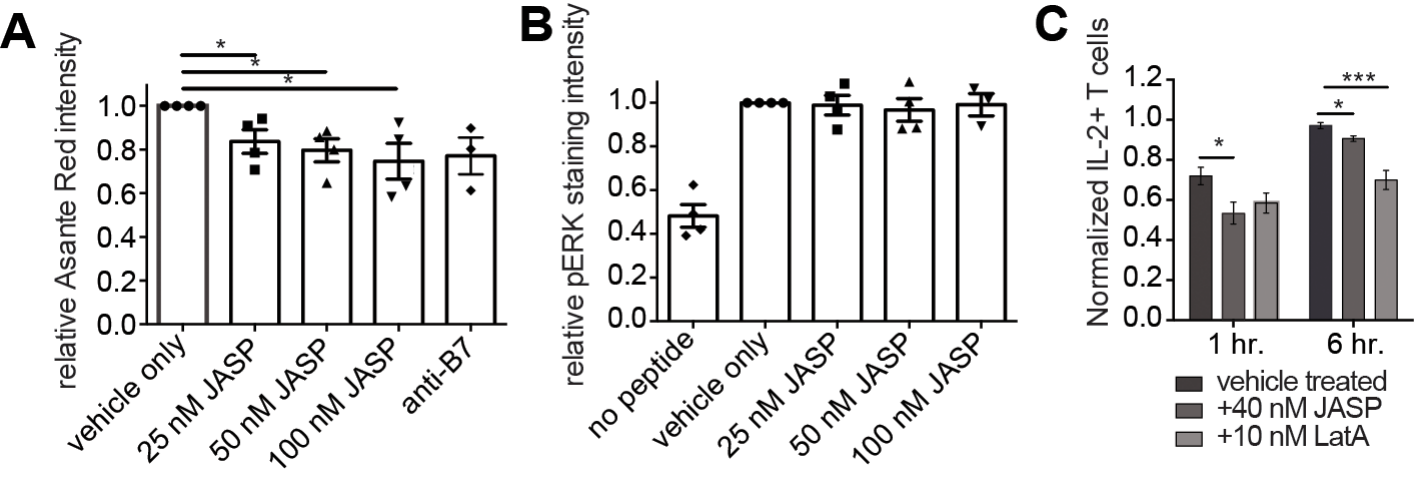
Jasplakinolide treatment selectively interferes with calcium signaling. **(A)** 5C.C7 T cells were activated with CH27 APCs (10μM MCC) with vehicle or 25–100nM Jasplakinolide as indicated. As a measure of the T cell intracellular calcium concentration the median T cell Asante Calcium Red fluorescence at 650nm emission over a 1min time window following cell coupling is given selectively in T cell:APC couples. Representative raw data including the gating strategy to identify cell couples and the effect of Jasplakinolide on cell coupling in the presence of agonist peptide and its absence as a negative control are given in S3A–S3C Fig. 4 experiments were analyzed. **(B)** 5C.C7 T cells were activated with CH27 APCs (10μM MCC) with vehicle or 25–100nM Jasplakinolide as indicated. Given is the mean fluorescence intensity of the staining for Erk1/2 T202/Y204 phosphorylation selectively in T cell:APC couples. Representative raw data including the gating strategy to identify cell couples and the effect of Jasplakinolide on cell coupling in the presence of agonist peptide and its absence as a negative control are given in S3D–S3F Fig. 4 experiments were analyzed. **(C)** IL-2 intracellular assays were performed with 5C.C7 T cells stimulated with CH27 APCs (10μM MCC) in the presence of 40nM Jasplakinolide or 10nM LatA and a DMSO control for 1 and 6 hours. The drugs were removed, a blocking MHC antibody was added and IL-2^+^ cells were measured at 24 hours. The percentage of IL-2^+^ cells is given normalized to the max percentage across the 1 and 6 hr. time points (3 independent experiments). Significance was determined by Student’s t-test and is indicated by asterisks (*p<0.05, ***p<0.0001).

Next we assessed general signaling outcomes by flow cytometry in 5C.C7 T cell:CH27 APC couples. Using emission of the calcium sensitive dye Asante Calcium Red at 650nm as a readout of T cell calcium signaling, Jasplakinolide treatment from 25–100nM significantly (p≤0.05) diminished T cell calcium signaling in T cell:APC couples to 84±6% to 75±6% of control (Fig 8A). This reduction is comparable to that seen upon costimulation blockade by pretreatment of APCs with antibodies against CD80 plus CD86 (75±10%). In contrast, T cell-driven Erk phosphorylation in 5C.C7 T cell:CH27 APC couples was not significantly diminished upon treatment with 25–100nM Jasplakinolide (Fig 8B). Thinning the early T cell actin matrix thus preferentially diminished calcium signaling. This is consistent with the loss of the lamellal localization of multiple calcium regulators upon low dose JASP treatment, as further discussed below. Alternate explanations however are also conceivable, e.g. a direct effect of Jasplakinolide on ER plasma membrane contact as required for store-mediated calcium entry [27, 28], even though Jasplakinolide concentrations used in those studies were at least 25-fold higher than the ones used here.

It is uncertain how closely the peak of signaling activity in the first five minutes of T cell activation controls long term effector function, such as cytokine secretion. Nevertheless, as effects of modest actin interference on long term T cell function are unclear, we determined whether IL-2 production was affected by 40nM Jasplakinolide. We treated T cell:APC cultures for 1 or 6h with 40nM Jasplakinolide, removed the drug and prevented further T cell stimulation with a MHC blocking antibody and assayed IL-2 after 24 hours. Treatment with 40nM Jasplakinolide only during the first hour of stimulation resulted in a subsequent 20±6% reduction (p = 0.02) in IL-2 positive T cells (Fig 8C). Because of the divergent time scales in the proximal signaling and IL-2 secretion experiments, these data do not allow conclusions on the role of lamellal actin on IL-2 secretion. However, an absence of an effect of 40nM Jasplakinolide on IL-2 secretion would have cast substantial doubt on the importance of lamellal signaling for the efficiency of T cell activation.

### Costimulation regulates lamellal localization

**Fig 9:**
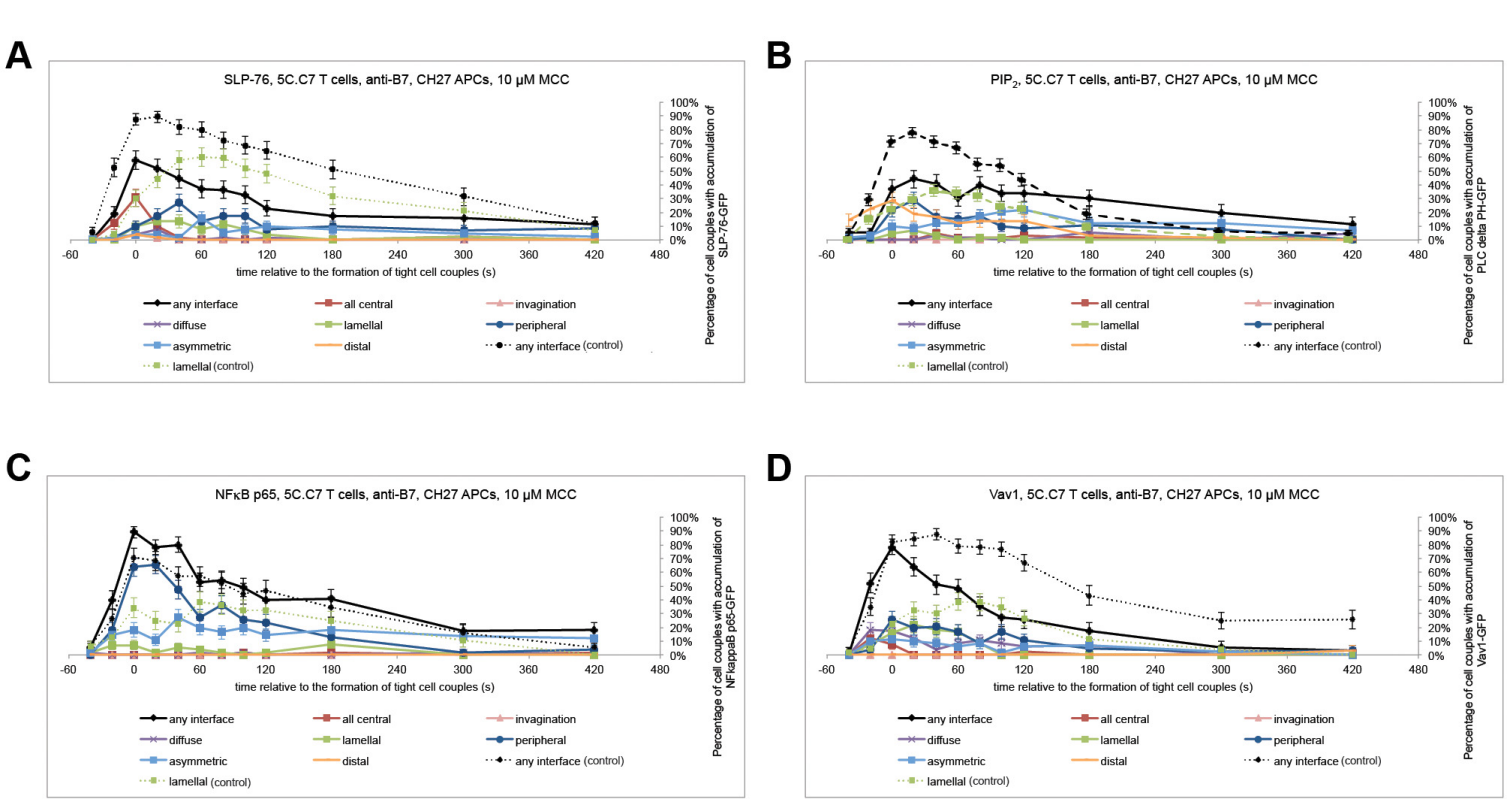
Costimulation blockade diminishes recruitment of signaling intermediates to the lamellal pattern. 5C.C7 T cells were activated by CH27 APCs and 10 μM MCC peptide upon costimulation blockade with 10 μg/ml anti-CD80 and anti-CD86. The percentage of T cells showing accumulation in defined patterns among all cell couples analyzed across multiple experiments are shown similar to Fig 5D for (**A**) SLP-76-GFP (number of cell couples analyzed across multiple independent experiments, n = 52) (**B**) PLCδPH-GFP (PIP_2_ sensor) (n = 59), (**C**) NFκB p65 (n = 55), and (**D**) Vav1-GFP (n = 51). Broken lines indicate interface accumulation under control conditions as a reference (Figs 1E, 1F and 4D of the accompanying manuscript and Fig 3C in [24]). Error bars are s.e.m..

For the further understanding of lamellal signaling, the identification of physiological regulators of lamellal localization is desirable. We therefore extended our investigation to CD28. Costimulation by CD28 enhances signaling efficiency and controls actin dynamics [11, 29–31]. Blocking the costimulatory ligands CD80 and CD86, the lamellal localization of all four signaling intermediates tested, SLP-76, PIP_2_, Vav1, and NFκB p65 was greatly diminished (Fig 9). For example, while 40 s after tight cell coupling, 58±7% of cell couples displayed lamellal SLP-76 localization under control conditions, this dropped to 13±5% upon costimulation blockade (p<0.001). Diminished lamellal accumulation is a prominent but not the only organization consequence of costimulation blockade. As shown previously, sustained (but less so early) central signaling is also impaired [3, 32], curiously a phenotype also matched by low dose JASP treatment (Fig 6). Interestingly, upon costimulation blockade calcium signaling was diminished to a comparable extent to direct interference with actin dynamics through the low dose JASP treatment (Fig 8A).

The specific elements of actin targeted by CD28 in T cell:APC couples are unresolved. To determine whether lamellal actin was impaired, we determined the extent of undulations of the T cell:APC interface by measuring interface length to diameter ratios as above. Similar to low dose JASP treatment, the increased undulations seen at the early time point in wild type T cells were lost upon costimulation blockade with interface length to diameter ratios at the early and late time points of 1.5±0.1 and 1.5±0.1 (Fig 2B), respectively. Many consequences of direct interference with actin dynamics using the low dose JASP treatment thus matched those upon costimulation blockade, arguing for a common signaling mechanism where a lamellal actin matrix enriches signaling intermediates to enhance their function. Nevertheless as to be expected for the complexity of system-wide signaling changes under different cellular activation conditions, modest differences could also be observed. In interface Vav1 accumulation for example, low dose JASP treatment only altered Vav1 patterning (Fig 5G) whereas costimulation blockade diminished overall interface Vav1 recruitment (Fig 9D).

A combination of lamellal phenotypes similar to low dose JASP treatment and costimulation blockade was also observed upon deficiency of the IL-2-inducible tyrosine kinase (Itk). Itk is critical for efficient T cell cytokine secretion [33] and effective actin accumulation at the T cell:APC interface [5, 34]. Lamellal accumulation of α-Pix, PIP_2_, Myosin1C and SLP-76 is dramatically impaired in Itk-deficient DO11.10 T cells [5], as is calcium signaling [35]. Similar to low dose JASP treatment and costimulation blockade, the increased membrane undulations seen in electron micrographs at the early time point in wild type T cells were lost upon Itk-deficiency with interface length to diameter ratios at the early and late time points of 1.5±0.1 and 1.4±0.1 (Fig 2B). The Itk data further support our suggestion of a common signaling mechanism where a lamellal actin matrix enriches signaling intermediates to enhance their function.

In summary, comparing low dose JASP treatment, costimulation blockade and Itk-deficiency lamellal actin, lamellal signaling localization, and calcium signaling were similarly impaired. First, this establishes costimulation and Itk as two physiological regulators of lamellal signaling localization. Second, these far-reaching though not complete similarities suggest that the enrichment of signaling intermediates in a lamellal actin matrix may be a common mechanism of signal amplification.

## Discussion

We have investigated the role of actin in signaling organization and function in the physiological activation of T cells by APCs. In T cell:APC couples T cell actin and signaling both display complex spatiotemporal distributions. Actin can form a ring around the periphery of the T cell:APC interface, underlies individual membrane extensions, cortical actin aligns with the plasma membrane, and a large transient actin sheet (‘lamellum’) extends from the undulating T cell:APC interface deep into the T cell (accompanying manuscript). Distinguishing molecular features of the various distributions still need to be determined. The spatiotemporal organization of T cell signaling was recently reviewed [24] and is extensively characterized in an accompanying manuscript. The interface center is enriched with proximal elements of T cell signaling as well as with core signaling focusing on the PKC pathway [1–3]. A lamellal distribution contains more distal signaling intermediates often involved in signal amplification. Various signaling intermediates, prominently adaptor proteins, provide a bi-directional connection by moving from the center to the lamellum over time and vice versa.

Given the complexity of subcellular organization, linking elements of actin dynamics to distinct signaling processes is difficult. Often, as is the case for T cells, molecular tools to target distinct subsets of cellular actin are lacking. Moreover, signaling distributions often align with multiple underlying cellular structures. For example, SLP-76 moves from the interface center to the lamellal pattern within the first minute of cell coupling and Vav localization has concurrent lamellal and peripheral features. The emergence of such complexity is a general feature of system studies. However, by revealing large-scale relations system scale studies also offer unique and substantial insight into complex processes that are inaccessible to single gene approaches: Using system scale investigation, one may find that a particular protein localization and signaling activity are related consistently for multiple signaling intermediates and across multiple cellular perturbations, even though such relations in the complex system signaling environment have to be preferential not absolute. For example in T cell activation, signaling intermediates associated with calcium signaling such as Vav1, SLP-76, and Itk were enriched in the lamellum and their loss from the lamellum under various T cell activation conditions, upon low dose JASP treatment, costimulation blockade or Itk deficiency, was consistently accompanied by diminished calcium signaling. Such conserved relations then suggest a functional connection. Importantly, the larger such relational networks are the more likely it becomes that localization and function are causally linked. In system data prevalent relations identify functional connections [36].

Here we have executed a large scale study to link changes in actin localization to ones in signaling distributions and activity. We found that a low concentration of Jasplakinolide preferentially affected spatiotemporally similar features of actin and signaling organization, the lamellal actin sheet and lamellal signaling localization. Importantly, lamellal localization coincides with the peak of proximal signaling and translocation of nuclear factor of activated T cells (NFAT) [3] and NFκB (accompanying manuscript) into the nucleus. Loss of lamellal localization was accompanied by the loss of the activity of SLP-76 as a lamellal signaling intermediate and diminished calcium signaling but not Erk phosphorylation as downstream signaling processes. The preferential impact on calcium signaling is consistent with previously established links between calcium signaling and actin [12] and, importantly here, with the distribution of upstream signaling intermediates: Key calcium regulators such as SLP-76 and Itk are prominently lamellal, signaling intermediates linked to RasGRP activation upstream of Erk phosphorylation such as DAG and PKCθ are central (accompanying manuscript). These data suggest a mechanism where recruitment of numerous signaling intermediates to an actin-based lamellum at the peak of T cell signaling activity enhances their activation and thus amplifies T cell signaling. Possible cellular details of this mechanism are discussed in the accompanying manuscript. Importantly, other elements of T cell organization are related to different signaling functions. For example, slowing the resolution of the large T cell invagination that forms at the interface center concurrently with the lamellal actin sheet by interfering with dynein light chain 1 activation diminishes LAT phosphorylation but leaves calcium signaling intact, thus affecting a different set of signaling intermediates as the low dose JASP treatment [20].

Costimulation blockade and Itk-deficiency caused loss of the early increase in membrane undulations as a hallmark of lamellal actin alongside impaired lamellal signaling localization and calcium signaling. This suggests that signal enhancement through lamellal localization with a focus on calcium signaling may be common.

## Materials and Methods

### Antibodies and Reagents

Antibodies for fixed cell microscopy were Alexa 488-α-SLP76 pY128 (BD Pharmingen) and α-LAT pY191 (Cell Signaling) with Alexa 488-goat α-rabbit IgG (Invitrogen). F-actin was stained with Alexa 633 or 488-Phalloidin (Invitrogen). CFSE and Cell Trace Violet were used as whole cell stains (Invitrogen). Additional antibodies for immunoblots were α-LAT pY191 (Cell Signaling), α-PKCθ pT538 (Cell Signaling), α-VAV1 pY174 (Sigma-Aldrich), and α-SLP76 pY128 (BD Pharmingen). Jasplakinolide and Latrunculin A were from Calbiochem.

### Mice, Cells and Drug Treatment

*In vitro* primed primary 5C.C7 T cells were set up from preferentially female 5C.C7 TCR transgenic mice of about 2 months of age, as previously described [3]. Costimulation blockade was executed as established [30]. Itk-deficient 5C.C7 mice were generated by breeding Itk-deficient B6 mice to 5C.C7 mice. The use of all mice has been reviewed and approved by the UT Southwestern IACUC committee and is covered by a University of Bristol Home Office license, respectively. For the low dose JASP treatment T cells were pretreated with 40nM Jasplakinolide (resuspended as a stock in DMSO and used at a 1:200 dilution of the stock to keep the final DMSO concentration at ≤0.5%) for 10min at 37°C and 40nM Jasplakinolide was maintained in the experiment. Latrunculin A was similarly used at 10nM.

### Microscopy and Image Analysis

As previously described in detail [3], T cells were transduced with MMLV-based retroviral particles to allow fluorescent sensor expression, transduced T cells were sorted for low GFP expression (2.6μM±0.4) to maximize physiological significance, and the interaction of sorted T cells with CH27 B cell lymphoma APCs loaded with 10μM moth cytochrome C peptide (fragment 88–103) was imaged at 37°C. Every 20 seconds a differential interference contrast image and 21 fluorescent z-planes spaced 1μm (total z volume = 20μm) were acquired with a CoolSnap HQ2 camera (Photometrics) and Metamorph (Molecular Devices) using a 40x (NA = 1.3) oil objective. Patterns of signaling sensor enrichment were assessed according to previously established quantitative criteria (Fig 2 in [3]). Briefly, the six, mutually exclusive interface patterns were: accumulation at the center of the T cell-APC interface (central), accumulation in a large T cell invagination (invagination), accumulation that covered the cell cortex across central and peripheral regions (diffuse), accumulation in a broad interface lamellum (lamellum), accumulation at the periphery of the interface (peripheral) or in smaller protrusions (asymmetric). Briefly, for each cell couple at each time point we first determined whether fluorescence intensity in the area of accumulation was >40% above the cellular fluorescence background. If so, the geometrical features of the area of accumulation, fraction of the interface covered, location within the interface, and extension of the area of accumulation away from the interface (Fig 2 in [3]), were used to assign the cell couple to one of the mutually exclusive patterns. The accumulation index was calculated as established [3, 37]. Briefly, the fraction of total cellular fluorescence translocated to an area of accumulation at the interface (defined as >40% above cellular fluorescence background) was multiplied by the fraction of cell couples analyzed that showed any interface accumulation.

For fixed cell imaging, CH27s were first adhered to a poly-L-lysine coated coverslip. T cells were then allowed to interact with APCs for 2 or 7min for early or late time points, respectively. T cells were fixed with 4% EM grade paraformaldehyde in PBS at 4°C for 20min and then stained for stimulated-emission depletion (STED) microscopy or deconvolution microscopy and mounted with ProLong Antifade (Invitrogen). For STED microscopy, T cells were stained with Alexa-488 conjugated Phalloidin or Alexa-488 conjugated α-pSLP-76 (Y128) and imaged as previously described in detail [38]. Briefly, cells were imaged through a 100×1.4 NA HCX APO objective on a Leica TCS STED CW system controlled by Leica AS AF software. Alexa Fluor 488 was excited using a 488nm Argon laser and STED depletion was achieved using a 592nm continuous wave fiber laser. For deconvolution microscopy in up to three colors a pDV Deltavision microscope (Applied Precision) equipped with an Olympus APO 40x oil objective (NA = 1.3) and Cool Snap HQ2 camera (Photometrics) was used. Image acquisition and deconvolution with a constrained iterative deconvolution algorithm were performed with softWoRx software v 2.0 (Applied Precision). A single DIC reference image and fluorescent z-stacks spanning the entire cell (0.2μm z-step) were acquired for each field. All image analysis for fixed cell microscopy was performed in Image J (NIH) as described below. Fixation of cell couples for microscopy required additional post acquisition analysis to assess the timing of cell coupling, as described in detail in an accompanying manuscript. Briefly, the shape of some T cells in the ‘late’ samples made it apparent that some cell couples had formed only briefly before fixation. We therefore used two morphological criteria for the post acquisition identification of ‘early’ cell couples, the presence of a uropod and T cell elongation: T cells with a distinct uropod (a T cell with an inversion of curvature of the plasma membrane at the distal pole) or a cell length (perpendicular to the interface) to interface diameter ratio of >1.25 were considered ‘early’ as both features were found almost exclusively during the first two minutes of live T cells coupling to APCs. Measurements of how deep actin and lamellal localized signaling intermediates reach into the T cell away from the interface were calculated using the box tool in Image J, either with a single box spanning the entire interface or with separate boxes for the interface center (inner 50% of the interface diameter) and periphery (outer 25%). The first box was at the interface, equal size boxes were then moved into the T cell in defined distance increments. Colocalization analysis for pSLP-76 and F-actin was performed with the JACoP plugin for Image J. Briefly, a binary mask for each channel was generated by linear thresholding and colocalization was assessed by calculation of the Pearson’s correlation coefficient (PCC) and Mander’s overlap coefficient. The distance of phosphorylated SLP-76 cluster from the interface was assessed by line scans and calculated from the point at which the APC fluorescence dropped to half-maximum. Phosphorylated SLP-76 clusters were evaluated with the Object Counter 3D plugin for Image J.

### Electron Microscopy

5C.C7 T cells ±40nM Jasplakinolide and peptide-loaded CH27s (10μM MCC) were centrifuged together for 30s at 350g to synchronize cell coupling, the cell pellet was immediately resuspended to minimize unspecific cell coupling and cellular deformation and the cell suspension was further incubated ±40nM Jasplakinolide at 37°C. After 2 and 5min for early and late time points, respectively, the cell suspension was high pressure frozen and freeze substituted to Epon as described previously [39]. Briefly, the Leica EM PACT2 with a Rapid Transfer System was used to high-pressure freeze T cell:APC suspensions. Frozen samples were substituted with 1% osmium tetroxide plus 0.1% uranyl acetate in acetone at -90°C, and subsequently embedded in Epon. Ultrathin sections were analyzed in a FEI Tecnai12 Biotwin equipped with a bottom-mount 4*4K EAGLE CCD camera. T cell:APC couples were identified in electron micrographs through their wide cellular interface. As described above and in more detail in an accompanying manuscript, the time point assignment of cell couples was filtered using morphological criteria post acquisition. Briefly, presence of a uropod and T cell elongation were used to identify early cell couples. In addition, cell couples with a distance of the nucleus from the cellular interface of more than 1μm were also classified as early (S4 Fig). Fulfillment of two of the three criteria was sufficient for time point assignment. The morphological filtering criteria remained largely consistent upon low dose JASP treatment. Supporting this notion, we have quantified the closest distance between the nucleus and the plasma membrane, under control conditions and upon low dose JASP treatment at early and late time points. We found that the approximation of the nucleus to the interface over time was not affected by the low dose JASP treatment (S4 Fig). To quantify the extent of membrane undulations, we measured the interface length in thin EM sections using Metamorph, i.e. the length of the undulating plasma membrane across the interface (red line in Fig 2A), and divided it by the interface diameter, i.e. a straight line across the interface.

### Fluorescent Recovery after Photobleaching

Individual T cell:CH27 conjugates were focused on and bleaching was done within the first 2min of cell conjugate formation. A pDVRT Deltavision deconvolution microscope (Applied Precision) equipped with a Quantitative laser module for FRAP, an Olympus APO 40x oil objective (NA = 1.3), and Cool Snap HQ2 camera (Photometrics) and controlled with Deltavision softWoRx software was used. All FRAP was performed at 37°C. Three prebleach images were acquired and then 10×10ms 488nm laser pulses (100% power) bleached a ~1μm Gaussian spot at the T cell:APC interface to near 50% of the prebleach intensity. Post bleach images were acquired every 255ms for a total of 30s to 2min depending on the protein. Analysis of recovery was performed manually in Image J by calculating the intensity in the bleach spot before and after bleaching. Background subtracted data was normalized to the average intensity of the 3 prebleached images and was fitted in Prism (Graphpad) with the equation Y_(f)_ = (Y_max_-Y_min_)(1-e^-kt^)+Y_min_. Y_(t)_ is the intensity of fluorescence at time t, Y_max_ and Y_min_ are the maximum and minimum intensities of fluorescence post-bleaching and k is the rate constant of recovery.

### Western blotting for phosphorylated signaling intermediates

Phosphorylation of SLP-76, Vav1, LAT, and PKCθ were determined by Western blotting analysis of cell extracts from T cell-APC couples, as previously described [25].

### T cell calcium signaling and Erk phosphorylation

For the determination of the elevation of the T cell intracellular calcium concentration, 5C.C7 T cells were loaded with 10 μM Asante Calcium Red acetoxymethly ester (Teflabs, Austin, TX). CH27 APCs incubated with 10 μM MCC agonist peptide were loaded with 20 μM CellTrace Violet (Molecular Probes). At 37°C in the presence of 10% serum, T cells were incubated with 2 x the indicated Jasplakinolide concentrations or DMSO as vehicle control for 10min, equal volumes of both cell types were mixed representing 3x10^6^ T cells and 10^6^ APCs, and spun for 30s at 350g to synchronize cell coupling. The cell pellet was immediately resuspended to minimize unspecific cell coupling and flow cytometry recording was started within 30s. After 4min Ionomycin was added to 1μg/ml. T cell:APC couples were identified as live events double positive for CellTrace Violet and Asante Calcium Red (S3A–S3C Fig). To determine calcium signaling the median fluorescence intensity of Asante Calcium Red at 650nm emission was measured in a 1min time window both after cell coupling and addition of Ionomycin. The increase in Asante Calcium Red fluorescence intensity in T cell:APC couples over that in non-conjugated T cells was normalized to that of vehicle control-treated T cell:APC couples.

To determine Erk1/2 phosphorylation at T202/Y204 in T cell:APC couples, 5C.C7 T cells and CH27 APC incubated with 10μM MCC agonist peptide were loaded with 2μM CFSE and 20μM CellTrace Violet, respectively. Cell couples were formed as in the calcium experiments and after 2min the cells were fixed and stained using the BD PhosFlow Fix 1 and Perm III reagents and an Alexa647-conjugated anti-Erk1/2 phospho-T202/Y204 antibody (BD). T cell:APC couples were identified as live cell events double positive for CellTrace Violet and Fluorescein (S3D–S3F Fig).

### IL-2 Intracellular Stains

Cell Trace Violet (Invitrogen)-labeled 5C.C7 T cells were mixed with peptide loaded CH27s at a ratio of 1:1. For experiments with actin drugs, Jasplakinolide (40nM) and Latrunculin A (10nM), 96 well round bottom plates were used and cells were mixed and centrifuged together to force interaction. The actin drugs were removed after the indicated time point and 10μg/mL α-I-E^k^ (BD Pharmingen) was added to block further T cell:CH27 interactions. The T cells were analyzed for intracellular IL-2 at 24h according to standard protocols.

### Statistical Analysis

To determine a significant change in spatiotemporal patterning, a proportion z-test was performed. Otherwise, statistical significance was determined with an unpaired Student’s *t*-test or 1-WAY ANOVA when appropriate. Statistical analysis was performed with GraphPad Prism (v5.0) or in some cases Excel.

## Supporting Information

S1 Fig Low dose JASP moderately interference with cell coupling and slows μm scale actin mobility. **(A)** 5C.C7 T cells were stimulated with peptide loaded CH27 APCs (10μM MCC) in the presence of 40nM or 100nM Jasplakinolide or buffer only, as indicated. The fraction of T cells contacting an APC that proceed to form a tight cell couple is given with the SEM. 3–10 independent experiments were analyzed per condition. **(B)**To directly assess μm-scale actin mobility upon low dose JASP treatment, we used fluorescence recovery after photobleaching (FRAP) in GFP-actin expressing T cells coupled to APCs. 5C.C7 T cells expressing GFP-actin were stimulated with peptide loaded CH27 APCs (10μM MCC) in the presence of 40nM Jasplakinolide (n = 41) or DMSO (n = 35). GFP-actin was bleached in a 1μm^2^ spot at either the interface periphery or in the lamellum and fluorescence recovery recorded. FRAP recovery curves and half times are given. Data are given separately for lamellal and peripheral actin as indicated. Significance was determined by Student’s t-test and is indicated by an asterisk (*p<0.05). Amongst the two prominent early actin patterns, lamellal and peripheral, peripheral actin was recovering faster under control conditions (t_1/2_ = 1.1±0.15s versus t_1/2_ = 1.9±0.2s, p<0.005) for unknown reasons. Upon low dose JASP treatment this faster component of actin dynamics was moderately slowed (t_1/2_ = 1.1±0.15s versus t_1/2_ = 1.55±0.1s, p = 0.02), consistent with minor F-actin stabilization. Lamellal actin recovery remained unchanged (t_1/2_ = 1.9±0.2s versus t_1/2_ = 1.85±0.15s). As the main conclusion of the FRAP experiments the modest reduction of actin mobility upon low dose JASP treatment is consistent with moderate F-actin stabilization by the low concentration of Jasplakinolide used. Mechanisms underlying more detailed observations of the FRAP analysis such as the slightly different size of the immobile fraction in peripheral versus lamellal actin or the preferential effect of the low dose JASP treatment on peripheral actin remain unresolved.
(TIF)

S2 Fig Disruption of actin by Jasplakinolide and Latrunculin A differentially affects T cell signaling. (**A-D)** Entire immunoblots with corresponding actin loading controls are given for Fig 7A–7D. (**E**) The pattern classification graph is given for 5C.C7 T cells expressing GFP-actin treated with 10nM LatA similar to Fig 1C (number of cell couples analyzed across multiple independent experiments, n = 27). (**F**) 5C.C7 T cells expressing GFP-actin were stimulated with peptide loaded CH27s (10μM MCC) treated with vehicle (DMSO, n = 25) or 10nM LatA (n = 25). The accumulation index measures the extent of interface accumulation, was calculated as described in the ‘Materials and Methods’, and is plotted relative to the time of tight cell conjugate formation. The control data are the same as in Fig 1F. (**G-J)** Entire immunoblots with corresponding actin loading controls are given for Fig 7E–7H.
(TIF)

S3 Fig Disruption of actin by Jasplakinolide selectively diminishes T cell calcium signaling. **(A,B)** Representative flow cytometry data in the determination of the elevation of T cell intracellular calcium concentration are given for 5C.C7 T cell:CH27 APC couples in the absence (A) or presence (B) of 10 μM MCC agonist peptide. On the left the gating strategy to identify non-conjugated T cells as CellTrace Violet (CTV) low events and T cell:APC couples as CellTrace Violet high/Asante Calcium Red high events is given. Percentage T cells only, B cells only (as CellTrace Violet high/Asante Calcium Red low events), and T/B cell couples are indicated. In the middle and on the right Asante Calcium Red emission at 650nm as a function of time is given for T cells only (middle) and T cell:APC couples (right). The induction of cell coupling precedes time 0, the short break between 250–300s indicates addition of Ionomycin at 1 μg/ml. **(C)** In the calcium flow cytometry experiments cell coupling was induced by a brief centrifugation step, as opposed to spontaneous cell coupling in the imaging experiments. To document the effect of Jasplakinolide on cell coupling under these conditions, the percentage of CellTrace Violet/Asante Calcium Red double-positive events is given normalized to control conditions (5.9±0.8% double-positives of all live cell events across all experiments). **(D,E)** Representative flow cytometry data in the determination of T202/Y204 Erk1/2 phosphorylation are given for 5C.C7 T cell:CH27 APC couples in the absence (C) or presence (D) of 10 μM MCC agonist peptide. On the left the gating strategy to identify non-conjugated T cells as CellTrace Violet (CTV) low events and T cell:APC couples as CellTrace Violet high/Fluorescein high events is given. Percentage T cells only, B cells only (as CellTrace Violet high/Fluorescein low events), and T/B cell couples are indicated. On the right the Alexa647 fluorescence is given for T cells only (red) and T cell:APC couples (blue) with the respective mean fluorescence intensity (MFI) indicated. Note that even the T cells in the T cell only gate may have briefly been in contact with APCs. **(F)** In the phospho-Erk flow cytometry experiments cell coupling was induced by a brief centrifugation step and followed by fixation at the end of the 2min T cell:APC incubation period, as opposed to spontaneous cell coupling in the imaging experiments. To document the effect of Jasplakinolide on cell coupling under these conditions, the percentage of CellTrace Violet/Fluorescein double-positive events is given as normalized to control conditions (17±1% double-positives of all intact cell events across all experiments).
(TIF)

S4 Fig Morphological criteria in the distinction between early and late time points–closest distance between the nucleus and the interface. 5C.C7 T cells were activated with CH27 APCs and 10 μM MCC agonist peptide upon control (number of cell couples analyzed across multiple independent experiments, n = 48) or low dose JASP (n = 35) treatment as indicated and processed for electron microscopy. The cell couples analyzed are the same as in Fig 2B. The closest distance between the nucleus and the T cell:APC interface is given. Error bars are s.e.m.
(TIF)

S1 Video Representative interactions of 5C.C7 T cells retrovirally transduced to express the indicated sensors with CH27 B cell lymphoma APCs in the presence of MCC agonist peptide (10 μM) are shown in S1 to S7 Videos. DIC images are shown on the top, with matching top-down, maximum projections of 3D sensor fluorescence data on the bottom. The sensor fluorescence intensity is displayed in a rainbow-like, false-color scale (increasing from blue to red). 20 s intervals in video acquisition are played back as 2 frames per second. The 5C.C7 T cell in S1 Video is transduced with GFP-actin and treated with 40nM Jasplakinolide. Cell coupling occurs in frame 7 (3s indicated video time). Persistent accumulation including early accumulation at the interface periphery is shown.
(MOV)

S2 Video The video is displayed similar to S1 Video. The 5C.C7 T cell in S2 Video is transduced with SLP-76-GFP and treated with 40nM Jasplakinolide. Cell coupling occurs in frame 5 (2s indicated video time). Rapid and very brief central accumulation is shown.
(MOV)

S3 Video The video is displayed similar to S1 Video. The 5C.C7 T cell in S3 Video is transduced with PLCδPH-GFP and treated with 40nM Jasplakinolide. Cell coupling occurs in frame 5 (2s indicated video time). Minimal accumulation is shown.
(MOV)

S4 Video The video is displayed similar to S1 Video. The 5C.C7 T cell in S4 Video is transduced with Themis-GFP and treated with 40nM Jasplakinolide. Cell coupling occurs in frame 6 (2s indicated video time). Minimal accumulation is shown.
(MOV)

S5 Video The video is displayed similar to S1 Video. The 5C.C7 T cell in S5 Video is transduced with Vav1-GFP and treated with 40nM Jasplakinolide. Cell coupling occurs in frame 8 (3s indicated video time). Rapid transition from central to lamellal accumulation that subsequently fades more slowly is shown.
(MOV)

S6 Video The video is displayed similar to S1 Video. The 5C.C7 T cells in S6 video are transduced with LAT-GFP and treated with 40nM Jasplakinolide. Two cell couples are shown with cell coupling occurring in frames 5/17 (left/right T cell) (2/7s indicated video time), respectively. Sustained predominantly central accumulation is shown.
(MOV)

S7 Video The video is displayed similar to S1 Video. The 5C.C7 T cell in S7 Video is transduced with PKCθ-GFP and treated with 40nM Jasplakinolide. Cell coupling occurs in frame 7 (3s indicated video time). Central accumulation, strong and transient early, weak late, is shown. Matching videos of 5C.C7 T cell/CH27 APC interactions without low dose JASP treatment are part of the accompanying manuscript and can also be accessed on the Wuelfing laboratory website at the University of Bristol at http://www.bristol.ac.uk/cellmolmed/research/infect-immune/wuelfing/spatiotemporal-patterning/.
(MOV)
