# Supplementary figures and images for: Modest Interference with Actin Dynamics in Primary T Cell Activation by Antigen Presenting Cells Preferentially Affects Lamellal Signaling
Source: PLoS One. 2015 Aug 3;10(8):e0133231. doi: 10.1371/journal.pone.0133231 (PMC4523178; doi:10.1371/journal.pone.0133231)

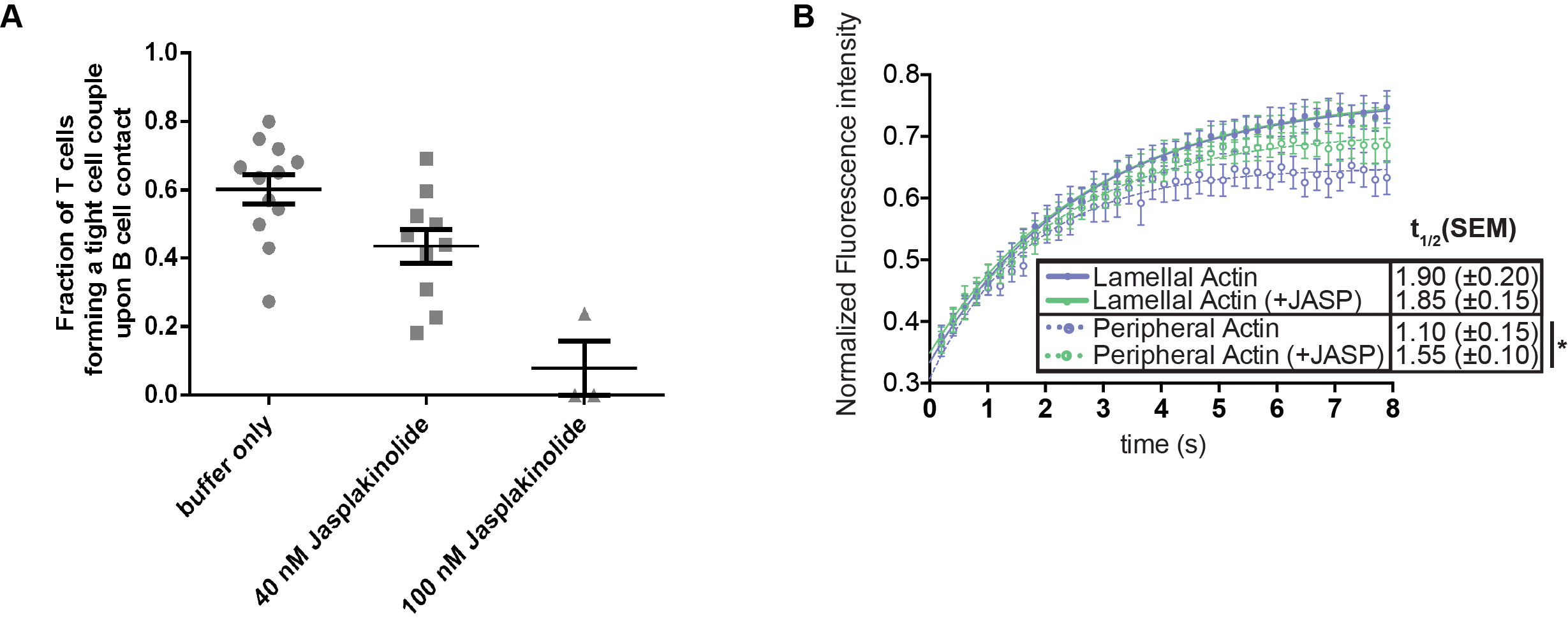

Supplement: S1 Fig — (A) 5C.C7 T cells were stimulated with peptide loaded CH27 APCs (10μM MCC) in the presence of 40nM or 100nM Jasplakinolide or buffer only, as indicated. The fraction of T cells contacting an APC that proceed to form a tight cell couple is given with the SEM. 3–10 independent experiments were analyzed per condition. (B)To directly assess μm-scale actin mobility upon low dose JASP treatment, we used fluorescence recovery after photobleaching (FRAP) in GFP-actin expressing T cells coupled to APCs. 5C.C7 T cells expressing GFP-actin were stimulated with peptide loaded CH27 APCs (10μM MCC) in the presence of 40nM Jasplakinolide (n = 41) or DMSO (n = 35). GFP-actin was bleached in a 1μm2 spot at either the interface periphery or in the lamellum and fluorescence recovery recorded. FRAP recovery curves and half times are given. Data are given separately for lamellal and peripheral actin as indicated. Significance was determined by Student’s t-test and is indicated by an asterisk (*p<0.05). Amongst the two prominent early actin patterns, lamellal and peripheral, peripheral actin was recovering faster under control conditions (t1/2 = 1.1±0.15s versus t1/2 = 1.9±0.2s, p<0.005) for unknown reasons. Upon low dose JASP treatment this faster component of actin dynamics was moderately slowed (t1/2 = 1.1±0.15s versus t1/2 = 1.55±0.1s, p = 0.02), consistent with minor F-actin stabilization. Lamellal actin recovery remained unchanged (t1/2 = 1.9±0.2s versus t1/2 = 1.85±0.15s). As the main conclusion of the FRAP experiments the modest reduction of actin mobility upon low dose JASP treatment is consistent with moderate F-actin stabilization by the low concentration of Jasplakinolide used. Mechanisms underlying more detailed observations of the FRAP analysis such as the slightly different size of the immobile fraction in peripheral versus lamellal actin or the preferential effect of the low dose JASP treatment on peripheral actin remain unresolved. (TIF) [file pone.0133231.s001.tif]

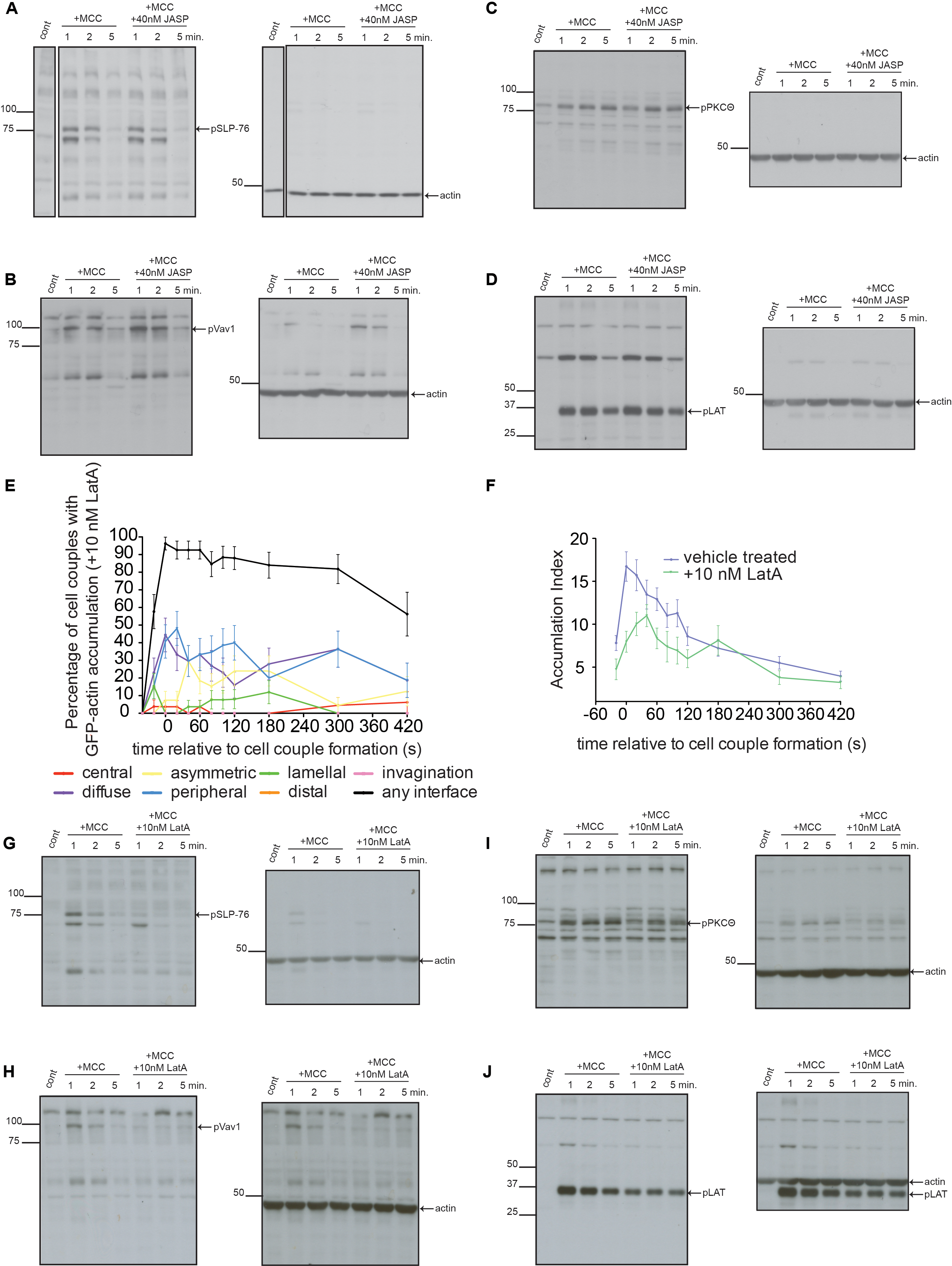

Supplement: S2 Fig — (A-D) Entire immunoblots with corresponding actin loading controls are given for Fig 7A–7D. (E) The pattern classification graph is given for 5C.C7 T cells expressing GFP-actin treated with 10nM LatA similar to Fig 1C (number of cell couples analyzed across multiple independent experiments, n = 27). (F) 5C.C7 T cells expressing GFP-actin were stimulated with peptide loaded CH27s (10μM MCC) treated with vehicle (DMSO, n = 25) or 10nM LatA (n = 25). The accumulation index measures the extent of interface accumulation, was calculated as described in the ‘Materials and Methods’, and is plotted relative to the time of tight cell conjugate formation. The control data are the same as in Fig 1F. (G-J) Entire immunoblots with corresponding actin loading controls are given for Fig 7E–7H. (TIF) [file pone.0133231.s002.tif]

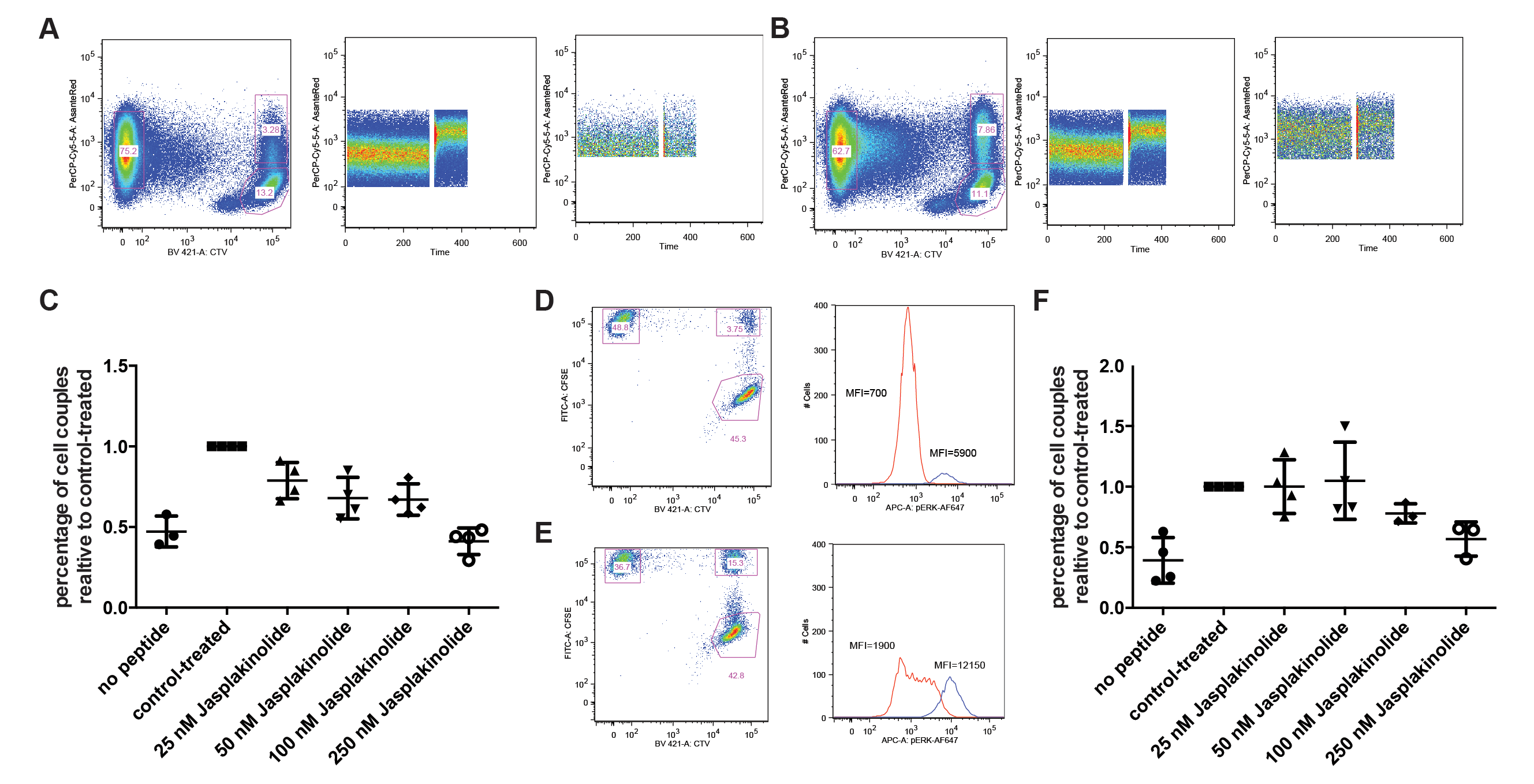

Supplement: S3 Fig — (A,B) Representative flow cytometry data in the determination of the elevation of T cell intracellular calcium concentration are given for 5C.C7 T cell:CH27 APC couples in the absence (A) or presence (B) of 10 μM MCC agonist peptide. On the left the gating strategy to identify non-conjugated T cells as CellTrace Violet (CTV) low events and T cell:APC couples as CellTrace Violet high/Asante Calcium Red high events is given. Percentage T cells only, B cells only (as CellTrace Violet high/Asante Calcium Red low events), and T/B cell couples are indicated. In the middle and on the right Asante Calcium Red emission at 650nm as a function of time is given for T cells only (middle) and T cell:APC couples (right). The induction of cell coupling precedes time 0, the short break between 250–300s indicates addition of Ionomycin at 1 μg/ml. (C) In the calcium flow cytometry experiments cell coupling was induced by a brief centrifugation step, as opposed to spontaneous cell coupling in the imaging experiments. To document the effect of Jasplakinolide on cell coupling under these conditions, the percentage of CellTrace Violet/Asante Calcium Red double-positive events is given normalized to control conditions (5.9±0.8% double-positives of all live cell events across all experiments). (D,E) Representative flow cytometry data in the determination of T202/Y204 Erk1/2 phosphorylation are given for 5C.C7 T cell:CH27 APC couples in the absence (C) or presence (D) of 10 μM MCC agonist peptide. On the left the gating strategy to identify non-conjugated T cells as CellTrace Violet (CTV) low events and T cell:APC couples as CellTrace Violet high/Fluorescein high events is given. Percentage T cells only, B cells only (as CellTrace Violet high/Fluorescein low events), and T/B cell couples are indicated. On the right the Alexa647 fluorescence is given for T cells only (red) and T cell:APC couples (blue) with the respective mean fluorescence intensity (MFI) indicated. Note that even the T cells [file pone.0133231.s003.tif]

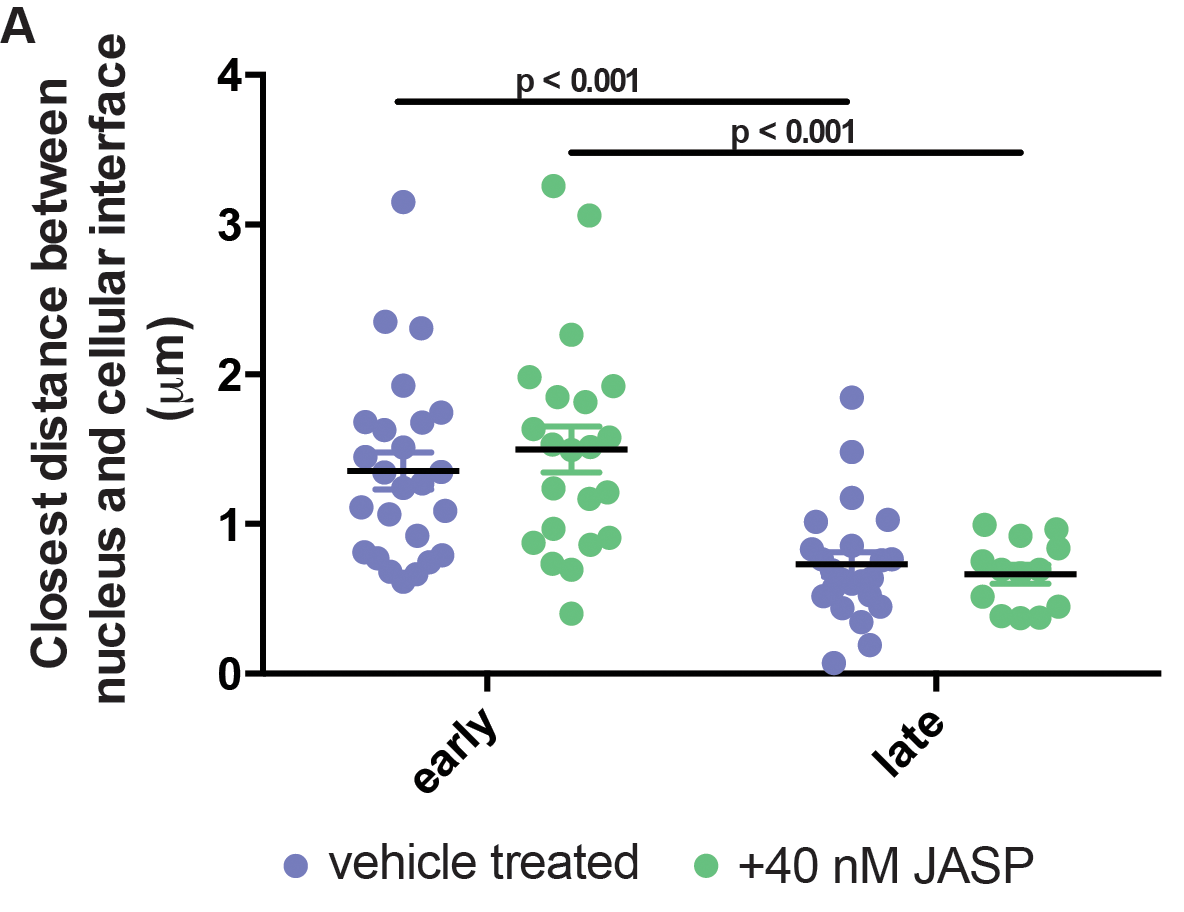

Supplement: S4 Fig — 5C.C7 T cells were activated with CH27 APCs and 10 μM MCC agonist peptide upon control (number of cell couples analyzed across multiple independent experiments, n = 48) or low dose JASP (n = 35) treatment as indicated and processed for electron microscopy. The cell couples analyzed are the same as in Fig 2B. The closest distance between the nucleus and the T cell:APC interface is given. Error bars are s.e.m. (TIF) [file pone.0133231.s004.tif]
